# CD200 ectodomain shedding into the tumor microenvironment leads to NK cell dysfunction and apoptosis

**DOI:** 10.1172/JCI150750

**Published:** 2022-11-01

**Authors:** Huw J. Morgan, Elise Rees, Simone Lanfredini, Kate A. Powell, Jasmine Gore, Alex Gibbs, Charlotte Lovatt, Gemma E. Davies, Carlotta Olivero, Boris Y. Shorning, Giusy Tornillo, Alex Tonks, Richard Darley, Eddie C.Y. Wang, Girish K. Patel

**Affiliations:** 1 European Cancer Stem Cell Research Institute, School of Biosciences,; 2 Department of Haematology, Division of Cancer & Genetics, School of Medicine, and; 3 Division of Infection and Immunity, School of Medicine, Cardiff University, Cardiff, United Kingdom.

**Keywords:** Oncology, Stem cells, Apoptosis, NK cells, Skin cancer

## Abstract

The basis of immune evasion, a hallmark of cancer, can differ even when cancers arise from one cell type such as in the human skin keratinocyte carcinomas: basal and squamous cell carcinoma. Here we showed that the basal cell carcinoma tumor–initiating cell surface protein CD200, through ectodomain shedding, was responsible for the near absence of NK cells within the basal cell carcinoma tumor microenvironment. In situ, CD200 underwent ectodomain shedding by metalloproteinases MMP3 and MMP11, which released biologically active soluble CD200 into the basal cell carcinoma microenvironment. CD200 bound its cognate receptor on NK cells to suppress MAPK pathway signaling that in turn blocked indirect (IFN-γ release) and direct cell killing. In addition, reduced ERK phosphorylation relinquished negative regulation of PPARγ-regulated gene transcription and led to membrane accumulation of the Fas/FADD death receptor and its ligand, FasL, which resulted in activation-induced apoptosis. Blocking CD200 inhibition of MAPK or PPARγ signaling restored NK cell survival and tumor cell killing, with relevance to many cancer types. Our results thus uncover a paradigm for CD200 as a potentially novel and targetable NK cell–specific immune checkpoint, which is responsible for NK cell–associated poor outcomes in many cancers.

## Graphical abstract

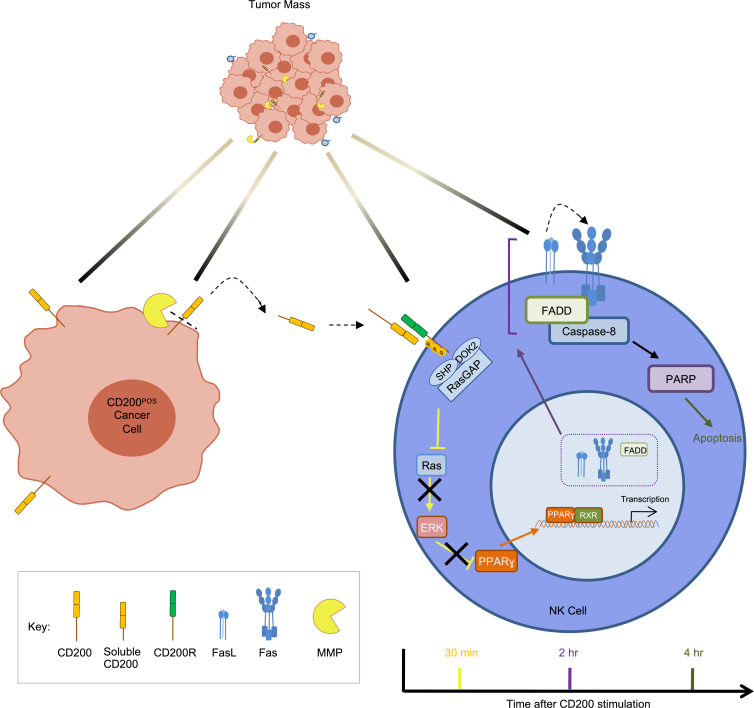

## Introduction

Epithelia are constantly exposed to environmental carcinogens and as such epithelial cancers, carcinomas, are the commonest adult malignancies and cancer-associated mortality. The transformation of epithelial tissues, carcinogenesis, is often a multistep process arising over many years, during which the cancer evolves to evade the immune system through immune editing (1).

As the largest organ, skin frequently develops cancers and in many countries skin cancer incidence eclipses the sum total of all other cancer types (2, 3). The 3 common skin cancer types account for over 95% of all skin cancers: malignant melanoma (MM), squamous cell carcinoma (SCC), and basal cell carcinoma (BCC). A hallmark of skin cancers is their high mutational burden and UV light mutational signature, as recently highlighted by genomic sequencing (4, 5). Although multiple driver mutations are necessary during SCC carcinogenesis, driver mutations that activate the MAPK and hedgehog growth factor pathways appear sufficient to promote MM and BCC, respectively (6, 7). Hence, targeted therapies for both MM and BCC that block the MAPK and hedgehog pathways have been developed and licensed, which greatly reduce the tumor burden but are rarely curative (8–12). In addition, characterization of immune evasion mechanisms in skin cancer has led to the identification of targetable immune checkpoints that are used to treat many cancer types, but with greatest success in MM and Merkel-cell carcinoma (13–15). Intriguingly, the 2 keratinocyte-derived carcinomas, SCC and BCC, have responded less favorably to both programmed death receptor 1 (PD-1) and cytotoxic T lymphocyte–associated protein 4 (CTLA-4) immune pathway checkpoint inhibition, despite their high mutational load, implying alternative mechanisms for immune evasion.

SCC has clinical and molecular hallmarks suggesting susceptibility to systemic immune therapy, including greater incidence among immunosuppressed transplant recipients, aging, UV light exposure, and high mutational burden (16–18). Indeed, an effective immune response is critical to control both SCC development and progression (19). The SCC tumor microenvironment (TME) is commonly associated with T cell infiltrates, which are overrepresented by central memory T cells that do not exhibit tumor-specific T cell receptor rearrangement (20). Moreover, approximately 50% are FOXP3^+^ regulatory T (Treg) cells that express interleukin 10 (IL-10) and transforming growth factor β (TGF-β) and reduced interferon γ (IFN-γ). Consistent with an abundance of Tregs, 85% of SCCs express the CTLA-4 ligand B7-H3, and clinical trials (ClinicalTrials.gov NCT04620200) with the CTLA-4 immune checkpoint inhibitor ipilimumab are ongoing (21). T cell activation is also regulated by PD-1 and is mediated by the interaction with its ligands, PD-L1 and PD-L2, with PD-L1 expression observed in 25% to 55% of primary human SCC cases and increasing to 70% in those associated with metastasis (21, 22). It has been shown that 25% and 50% of SCC patients with advanced locally unresectable or metastatic SCC respond to PD-1 immune checkpoint inhibitors pembrolizumab and cemiplimab, respectively (23, 24).

As with SCC, multiple lines of evidence point to the ability of the host immune system to eradicate BCC. The incidence of BCC is greater among immunosuppressed individuals, while cessation of immunosuppressive therapy in transplant recipients can reduce BCC occurrence (25–27). The BCC TME contains substantial numbers of tumor-infiltrating inflammatory cells (CD45^+^), representing 13.81% ± 10.84% (*n* = 21) of all cells (28). The BCC immune response consists of mainly T cells; both CD4^+^ and CD8^+^ T cells are present in the peritumoral infiltrate in BCC with a CD4^+^/CD8^+^ cell ratio of 2:1 (29). Most T cells also express the activation markers IL-2 receptor (CD25), CD45RO, and the transferrin receptor. BCC keratinocytes release the chemokine CCL22 to directly recruit Tregs that express the cognate receptor CCR4 (30). Relative to the surrounding normal skin, BCC tumor tissues contain significantly greater numbers of Tregs, greater than 25% of tumor-associated CD4^+^ T cells, similar to many cancers, including human SCC (30). Treg- and BCC keratinocyte–derived IL-10 and TGF-β released into the TME attenuates dendritic cell (DC) and T cell effector responses (31). IL-10 and the relative lack of IFN-γ, TNF-α, IL-2, and IL-12 in the BCC TME also hinders NK cell recruitment (29, 32). In 20% of BCC, the antitumor immune infiltrate is sufficient to promote histological regression (33). Local immune stimulation by application of immunogens, including licensed treatment with the TLR-7 and -8 agonist imiquimod, or cytokine injection facilitates tumor rejection (34–36). PD-L1 expression is observed in 22%–90% of primary human BCC cases, but in locally advanced or metastatic cases, PD-L1 expression was not enhanced (37–39). Although there are a number of trials ongoing, a proof-of-principal open-label study involving 16 cases of advanced BCC treated with pembrolizumab demonstrated an overall response rate of 38% (39).

To understand the basis for skin cancer immune evasion it is also important to consider the role of tumor-initiating cancer stem cells in skin cancer growth, which has been established for SCC and BCC but remains controversial in MM (28, 40–43). Consistent with the hierarchical growth pattern of epithelial tissues, including the skin (44–47), our group has identified small tumor-initiating cell subpopulations in both keratinocyte carcinomas using a classical in vivo approach (28, 40). Having established a robust in vivo model, we determined that within CD133^+^ SCC cells the tumor-initiating capacity was 1 cell per 400 cells, which represented approximately 1% of the tumor cells, compared with unsorted SCC cells which was 1 cell per 1 × 10^6^ cells (40). Consistent with a hair follicle adult tissue stem cell of origin, 1% to 3% of BCC cells expressed CD200 (OX-2), which demonstrates a 1500-fold tumor-initiating-cell enrichment compared with unsorted cells (28). CD200 is a 45 kDa transmembrane immunoregulatory protein of 278 amino acids (aa), which includes a 30 aa signal sequence, a 202 aa extracellular domain, a 27 aa transmembrane segment, and a 19 aa cytoplasmic domain (48, 49). It is highly conserved between species, with mouse and rat CD200 exhibiting 76% homology, even though it is widely but not ubiquitously expressed, underpinning its importance. Its receptor, CD200R, is restricted to both innate and acquired immune cells and upon receptor-ligand contact initiates a unidirectional inhibitory signal (50). Thus, the broad tissue distribution of CD200 and the immunologically restricted expression of CD200R are consistent with CD200 possessing an immunoregulatory function. Hence, for keratinocyte carcinoma, it can be hypothesized that immune evasion is paramount for tumor-initiating-cell survival in order to sustain tumor growth despite their differences. Herein, we have sought to compare keratinocyte TME immune cell profiles to elucidate potentially novel cancer immune evasion mechanisms.

## Results

### Characterization of the BCC TME.

**Figure 1:**
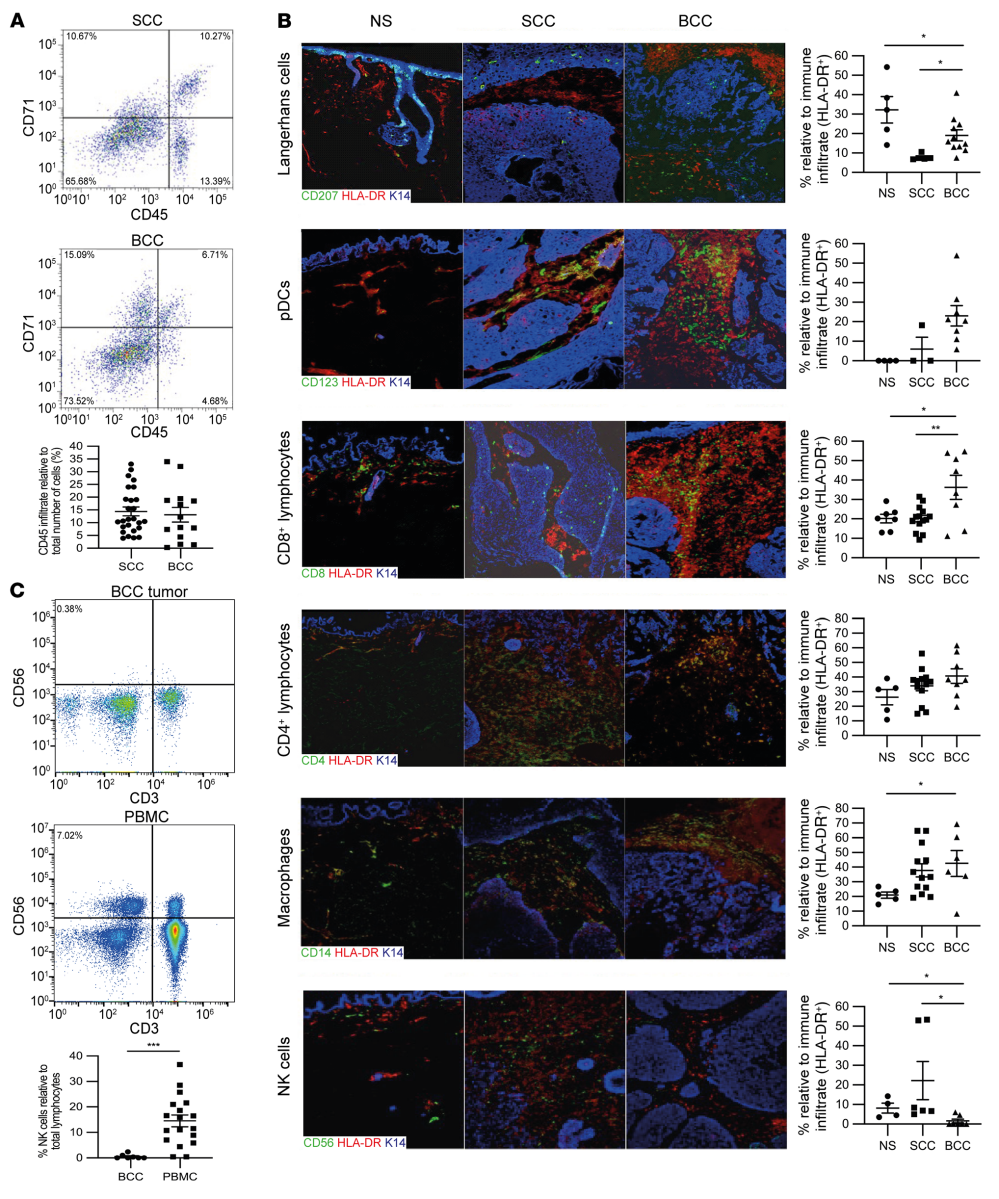
Characterization of skin tumor immune responses. (**A**) Frequency of immune cells (CD45^+^) in primary human BCC (*n* = 14) and SCC (*n* = 26) determined by flow cytometric analysis. (**B**) Determination of individual immune cell populations within normal skin (NS), SCC, and BCC by immunofluorescent labeling with antibodies against CD207 (Langerhans cells), CD123 (plasmacytoid dendritic cells, pDCs), CD8 (cytotoxic T lymphocytes), CD4 (helper T lymphocytes), CD14 (macrophages), and CD56 (NK cells). (**C**) Flow cytometric determination of NK cell frequency in primary human BCC tumor (*n* = 18) and matched patient PBMCs (*n* = 7). Data show mean ± SEM. All scale bars: 100 μm. The 2-tailed Student’s *t* test was used to determine the difference between BCC and SCC and between BCC and PBMC. One-way ANOVA was used to determine the difference between BCC, SCC, and NS. Data are presented as mean ± SD. *P < 0.05; **P < 0.01; ***P < 0.001.

The high mutational burden exhibited by keratinocyte carcinomas renders them susceptible to continuous immune editing. As expected, immune cells expressing the common leucocyte antigen (CD45) were prevalent within the BCC (13.11% ± 2.88% [*n* = 14]) and SCC (14.42% ± 1.73% ([*n* = 26]) TME (Figure 1A), consistent with antitumor immunity (Figure 1B). Within the tumor and surrounding tissue, antigen-presenting cells were evident, including Langerhans cells (CD207^+^HLA-DR^+^) (BCC, 19.08% ± 2.83% [*n* = 11] and SCC, 7.85% ± 0.69% [*n* = 5]), but less than in normal skin (32.22% ± 6.89% [*n* = 5]). CD123^+^HLA-DR^+^ plasmacytoid DCs (pDCs) were more prevalent in BCC than SCC and normal skin: BCC (23.04% ± 5.24% [*n* = 8]), SCC (6.07% ± 6.07% [*n* = 3]), and normal skin (0.00% ± 0.00% [*n* = 4]). These pDCs expressed the lymph node–homing chemokine receptor CCR7 (data not shown), which was not evident in normal skin, consistent with ongoing tumor-antigen presentation.

Consistent with skin tumor immunity, cytotoxic T lymphocyte (CD8^+^HLA-DR^+^) frequency was greater in BCC (36.23% ± 6.22% [*n* = 8]) than that in SCC (20.16% ± 1.70% [*P* < 0.01, *n* = 14]) and normal skin (20.14% ± 2.17% [*P* < 0.05, *n* = 7]). Likewise, T helper cell (CD4^+^HLA-DR^+^) frequency was also greater in BCC (40.68% ± 5.05% [*n* = 8]) than in SCC (33.96% ± 3.25% [*n* = 13]) and normal skin (26.30% ± 5.23% [*n* = 5]). Skin-resident macrophages (CD14^+^HLA-DR^+^) were greater in BCC (42.54% ± 8.83% [*n* = 6]) than normal skin (20.94% ± 2.10% [*P* < 0.05, *n* = 5]), but were also evident within SCC (37.75% ± 4.50% [*n* = 13]). In stark contrast, there was near absence of NK cells (CD56^+^HLA-DR^+^) in BCC (1.53% ± 1.00% [*n* = 7]) compared with SCC (22.21% ± 9.80% [*P* < 0.01, *n* = 6]) and normal skin (8.17% ± 2.55% [*P* < 0.05, *n* = 4]). Flow cytometric analysis of dissociated primary BCC from differing body sites confirmed near absence of NK cells within the BCC microenvironment (0.56% ± 0.34% [*n* = 7]), even though normal NK cell numbers were found to be present in matched patient blood samples (14.54% ± 2.31% [*P* < 0.01, *n* = 18]) (Figure 1C). In summary, the BCC TME uniformly demonstrated a selective absence of tumor-infiltrating NK cells.

### CD200 expression blocks NK cell killing activity.

**Figure 2:**
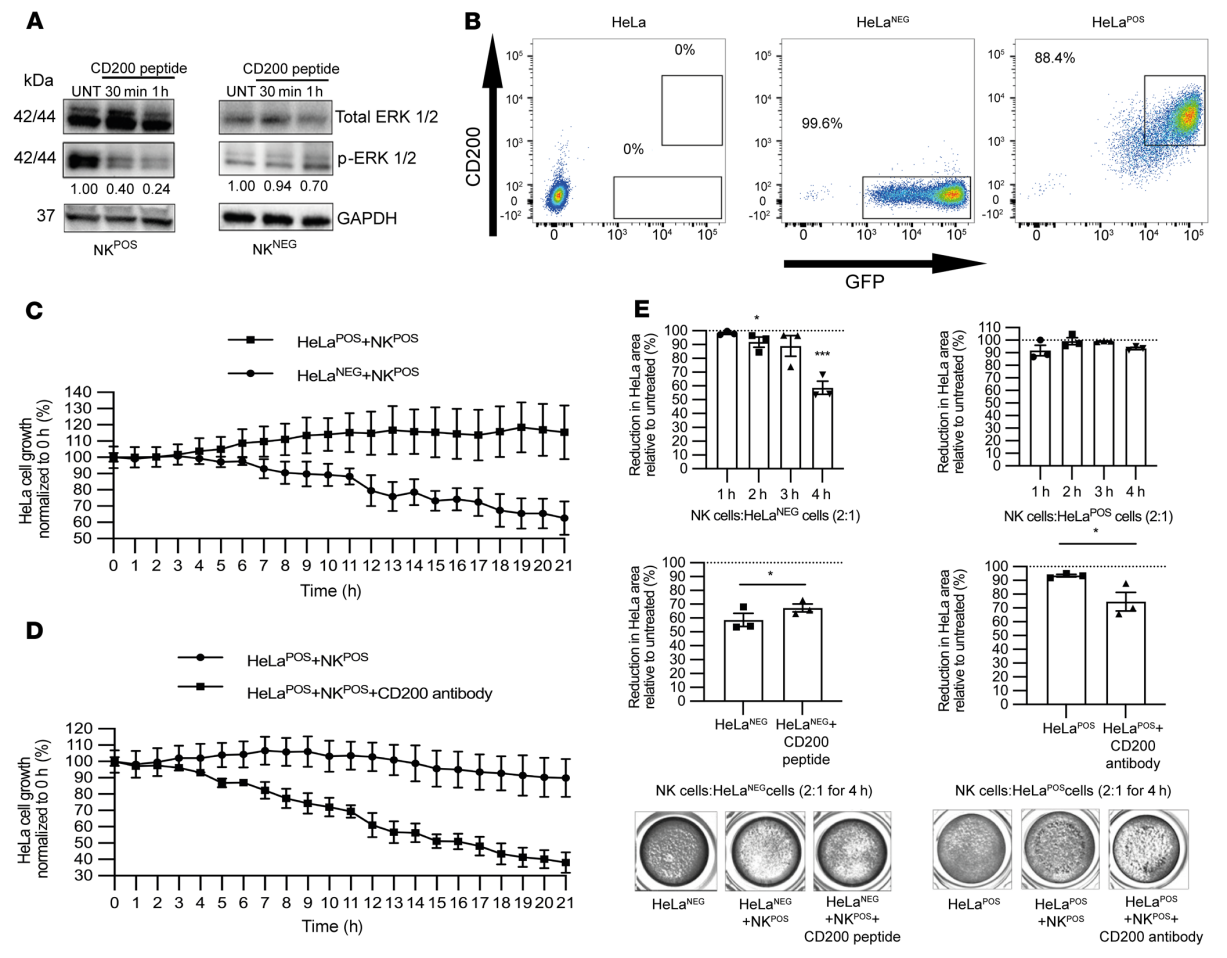
CD200 blocks NK cell killing. (**A**) NK^POS^ and NK^NEG^ cells were stimulated with a CD200 peptide (4 μg/10^6^ cells) for 30 minutes and 1 hour and activation of ERK was detected by immunoblotting whole-cell lysates for phosphorylated ERK1 and 2 relative to GAPDH. (**B**) Live cell flow cytometric analysis of HeLa cells transduced with a bicistronic GFP plasmid with and without CD200 lentivirus construct. (**C**) Time-lapse quantification of viable GFP-HeLa (target cell) cells in coculture with NK^POS^ (effector) cells at an E:T ratio of 5:1 (*n* = 3) over 20 hours; a significant difference was observed after 6 hours (*P* < 0.01). (**D**) Addition of a CD200-blocking antibody to HeLa^POS^-NK^POS^ cocultures restored HeLa cell killing compared with untreated cells after 6 hours (*P* < 0.01, *n* = 3). (**E**) Coculture in 24-well plate at an E:T ratio of 2:1 (3 replicates), in which adherent viable cells were stained and quantified over 1 to 4 hours, with and without CD200 peptide or CD200-blocking antibody, as shown in bottom panels. Data are presented as mean ± SD of 3 independent experiments. *P < 0.05; ***P < 0.001 by 2-tailed Student’s *t* test.

In acute myeloid leukemia, CD200 expression led to impaired NK cell killing. We therefore sought to determine whether CD200 expression on epithelial cells could also mediate this NK cell immune evasion. Since CD200R is expressed on a restricted population of NK cells, we first identified NK cell lines that expressed CD200R (NK92MI, herein referred to as NK^POS^) and CD200R negative (NKL, herein referred to as NK^NEG^) (Supplemental Figure 1A; supplemental material available online with this article; https://doi.org/10.1172/JCI150750DS1). Incubation with CD200 peptide led to a 4-fold reduction in p-ERK1/2 levels, consistent with CD200 signaling through activation of the MAPK pathway and as an indicator of NK cell activation, in NK^POS^ but not NK^NEG^ cells, within 60 minutes (Figure 2A). Furthermore, when human CD200 peptide was incubated with CD200R-positive murine neuronal cells there was also a reduction in p-ERK1/2 levels relative to total ERK, since the extracellular portion of human and mouse CD200R share 86% amino acid sequence homology (48) (Supplemental Figure 1, B and C). Hence, the membrane-bound CD200 and CD200 peptide similarly led to a reduction in MAPK signaling within NK^POS^ cells.

To determine whether CD200 expression on epithelial tumor cells could block NK cell killing activity, we transduced the cervical keratinocyte cancer cell line HeLa, which does not normally express CD200, with a bicistronic GFP plasmid with and without CD200 (hereafter called HeLa^POS^ and HeLa^NEG^, respectively) and confirmed cell membrane expression by flow cytometry (Figure 2B). In an Incucyte time-lapse coculture assay (with NK^POS^ cells), which enumerated individual transduced HeLa cells through GFP fluorescence levels, we observed a consistently greater reduction in HeLa^NEG^ compared with HeLa^POS^ cell numbers (*P* < 0.05; Figure 2C and Supplemental Video 1). The findings were similar when NK^POS^ cells were cocultured with the cutaneous keratinocyte cell line HaCaT transduced with a bicistronic GFP plasmid with and without CD200 (*n* = 4; Supplemental Figure 1D). However, killing could be restored in CD200-expressing HaCaT cells if cultured with NK^POS^ cells pretreated with CD200R shRNA (Supplemental Figure 1E), confirming that NK activity was dependent on CD200 ligand–receptor interaction. Increasing the ratio of NK^POS^ cells to transduced HeLa cells led to greater killing, but HeLa^POS^ cells consistently demonstrated less cell death (Supplemental Figure 1F). However, HeLa^POS^ killing by NK^POS^ cells was reversed by addition of a CD200-blocking antibody (*P* < 0.05; Figure 2D). Reproducibly, 4 hours after coculture with NK^POS^ cells, we observed a significant reduction in HeLa^NEG^ confluence compared with baseline (*P* < 0.01) and compared with HeLa^POS^ (*P* < 0.01; Figure 2E and Supplemental Figure 2A). Addition of CD200 peptide to HeLa^NEG^ and NK^POS^ coculture reduced killing (*P* < 0.05), while addition of CD200-blocking antibody to HeLa^POS^ and NK^POS^ coculture led to increased killing (*P* < 0.05). Thus, epithelial tumor cell CD200 expression hindered NK cell killing.

**Figure 3:**
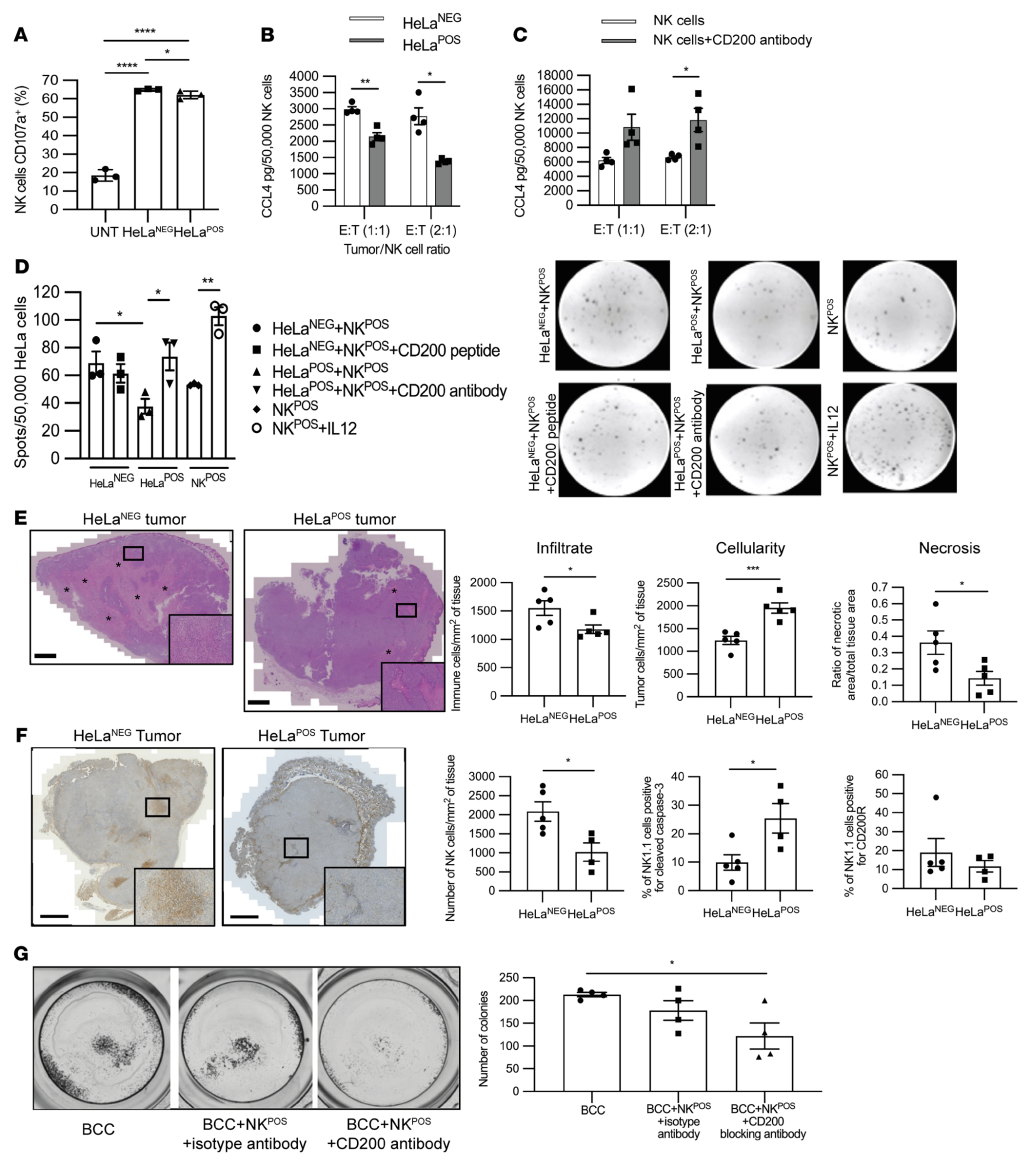
CD200 blocks NK cell activation, degranulation, and cytokine release. (**A**) Flow cytometric analysis histogram of CD107a expression levels on CD56^+^ NK^POS^ cells cocultured with HeLa^POS^ or HeLa^NEG^ at an E:T ratio of 5:1 for 4 hours. (**B** and **C**) CCL4 ELISA of culture supernatant from NK^POS^ cells cocultured with HeLa^POS^ or HeLa^NEG^ at an E:T ratio of 1:1 and 2:1 for 4 hours (**B**), and together with CD200-blocking antibody (**C**). (**D**) ELISpot IFN-γ determination from NK^POS^ cells cocultured with HeLa^POS^ or HeLa^NEG^ at an E:T ratio of 1:10 for 4 hours, together with either CD200 peptide or CD200-blocking antibody. (**E** and **F**) Day 5 tumors from nude mice were grafted with 1 × 10^6^ HeLa^POS^ or HeLa^NEG^ (*n* = 5 each). Histological analysis of tumor cellularity, necrosis and inflammatory cell infiltration (**E**), and paraffin-embedded sections labeled with anti-NK1.1, anti–cleaved caspase 3, and anti-CD200R antibodies by immunohistochemistry to determine NK cell infiltrate, using spleen sections as positive control, and frequency of cleaved caspase 3–positive and CD200R-positive cells (**F**). Asterisks show necrotic tissue area. (**G**) BCC colonies were established in primary culture (*n* = 3) over a period of 2 weeks using an irradiated NIH/3T3 mouse fibroblast layer. Colonies were coincubated with NK^POS^ cells and treated with either a CD200-blocking antibody or an isotype antibody control for 4 hours and then enumerated. Data are presented as mean ± SD of 3 independent experiments. *P < 0.05; **P < 0.01; ***P < 0.001; ****P < 0.0001 by 2-tailed Student’s *t* test (**A**–**D**) or 2-way ANOVA with Bonferroni’s post hoc test (**E**–**G**).

To understand how CD200 expression blocked NK cell killing, we next assessed the effect of CD200 on NK^POS^ cells to determine release of cytotoxic granules, chemokines, and cytokines. The release of cytotoxic granules, including granzymes and perforins, is associated with degranulation-related cell surface expression of the lysosomal membrane protein Lamp1 (CD107a) (32). Flow cytometric analysis of NK^POS^ cells stimulated with PMA/ionomycin and treated with monensin led to a 3-fold increase in CD107a expression compared with baseline (*P* < 0.001; Supplemental Figure 2, B and C). Similarly, coculture of NK^POS^ cells together with either HeLa^NEG^ or HeLa^POS^ cells also led to increased intracellular p-ERK and cell surface CD107a expression over baseline, more so in HeLa^NEG^ than HeLa^POS^ cells (*P* < 0.0001; Supplemental Figure 2D and Figure 3A). To effect killing, NK cells also release both chemokines and cytokines, notably CCL4 and IFN-γ (51). HeLa^POS^ and HeLa^NEG^ cells were coincubated as before with NK^POS^ cells and the supernatant was analyzed for CCL4 by ELISA. CCL4 levels in supernatant were lower in HeLa^POS^ compared with HeLa^NEG^ cocultures at effector/target (E:T) ratios of 1:1 (*P* < 0.01) and 2:1 (*P* < 0.05) (*n* = 4; Figure 3B). Addition of the CD200-blocking antibody to the HeLa^POS^ and NK^POS^ cell coculture led to a 2-fold increase in CCL4 secretion, reaching significance at a 2:1 ratio (*P* < 0.05; Figure 3C). HeLa^POS^ or HeLa^NEG^ and NK^POS^ cell coculture IFN-γ release was assessed by ELISpot assay with an E:T ratio of 1:10 to ensure individual spots could be enumerated. There was a significant increase in IFN-γ secretion within the HeLa^NEG^-NK^POS^ coculture when compared with the HeLa^POS^-NK^POS^ coculture (*P* < 0.05; Figure 3D). HeLa^POS^-NK^POS^ coculture IFN-γ secretion was similar to that of NK^POS^ cells alone but increased significantly when CD200-blocking antibody was added to the coculture (*P* < 0.05). HeLa^NEG^-NK^POS^ cell coculture IFN-γ secretion levels were reduced in the presence of CD200 peptide, although this did not reach significance (Figure 3D) compared with the significant increase in IFN-γ secretion levels following addition of IL-12 (*P* < 0.01; Figure 3D). In conclusion, CD200 signaling led to a reduction in NK cell activation, degranulation, and chemokine and cytokine release.

Since human CD200 peptide induced murine CD200R signaling, we were able to determine the effect of CD200 expression on the inflammatory infiltrate within grafted tumor cells. HeLa cells grow rapidly and reproducibly to form tumors when implanted into nude mice, which retain NK cells. We grafted 1 × 10^6^ HeLa^POS^ or HeLa^NEG^ cells into the flank of nude mice (*n* = 5/cell type) and after only 5 days, as expected, we observed no difference in tumor volume (data not shown). Quantification of H&E-stained sections showed that HeLa^NEG^ compared with HeLa^POS^ tumors demonstrated reduced number (cellularity) of tumor cells (1,240 ± 90.99 vs. 1,952 ± 114.80 per mm^2^ of tissue; *P* < 0.001), with a greater ratio of necrotic to normal tumor area (0.36 ± 0.07 vs. 0.14 ± 0.04; *P* < 0.05), and greater immune cell infiltrate (1,551 ± 128.60 vs. 1,180 ± 77.39 per mm^2^ of tissue; *P* < 0.05) (Figure 3E). In vivo, HeLa^POS^ but not HeLa^NEG^ tumors demonstrated CD200 expression (Supplemental Figure 3). Tumor sections labeled with anti–mouse NK1.1 antibody demonstrated reduced NK cell infiltrate surrounding HeLa^POS^ tumors when compared with HeLa^NEG^ tumors (1,024 ± 239.80 vs. 2,085 ± 251.90 NK cells/mm^2^ of tumor tissue, respectively; *P* < 0.05; Figure 3F and Supplemental Figure 3, A and B). Furthermore, the percentage of NK1.1 cells positive for cleaved caspase 3 was higher in HeLa^POS^ tumors when compared with HeLa^NEG^ tumors (*P* < 0.05; Figure 3F). The findings were similar when the human cutaneous BCC cell line, UWBCC1, transduced with a bicistronic GFP plasmid with and without CD200 (UWBCC1^POS^ and UWBCC1^NEG^, respectively), was xenografted (*n* = 10; Supplemental Figure 3C). Thus, keratinocyte carcinoma CD200 expression, whether in cervical carcinoma or BCC, promotes tumor growth.

We next determined whether blocking CD200 signaling facilitates NK cell–mediated BCC killing. Similar numbers of human BCC colonies were established in primary culture (*n* = 3 different tumors) in triplicate over a period of 2 weeks using a method previously published by our lab (28). Enumerated colonies were then coincubated with NK^POS^ cells alone or together with either a CD200-blocking antibody or an isotype antibody control for 4 hours. There was a small reduction in colonies after the addition of NK^POS^ cells with the isotype, but this did not reach significance. However, there was a 50% reduction in colony numbers following coincubation with NK^POS^ cells that were simultaneously treated with the CD200-blocking antibody (*P* < 0.05; Figure 3G). In conclusion, NK cells could kill BCC cells upon blocking CD200 signaling.

### CD200 induced NK cell apoptosis.

**Figure 4:**
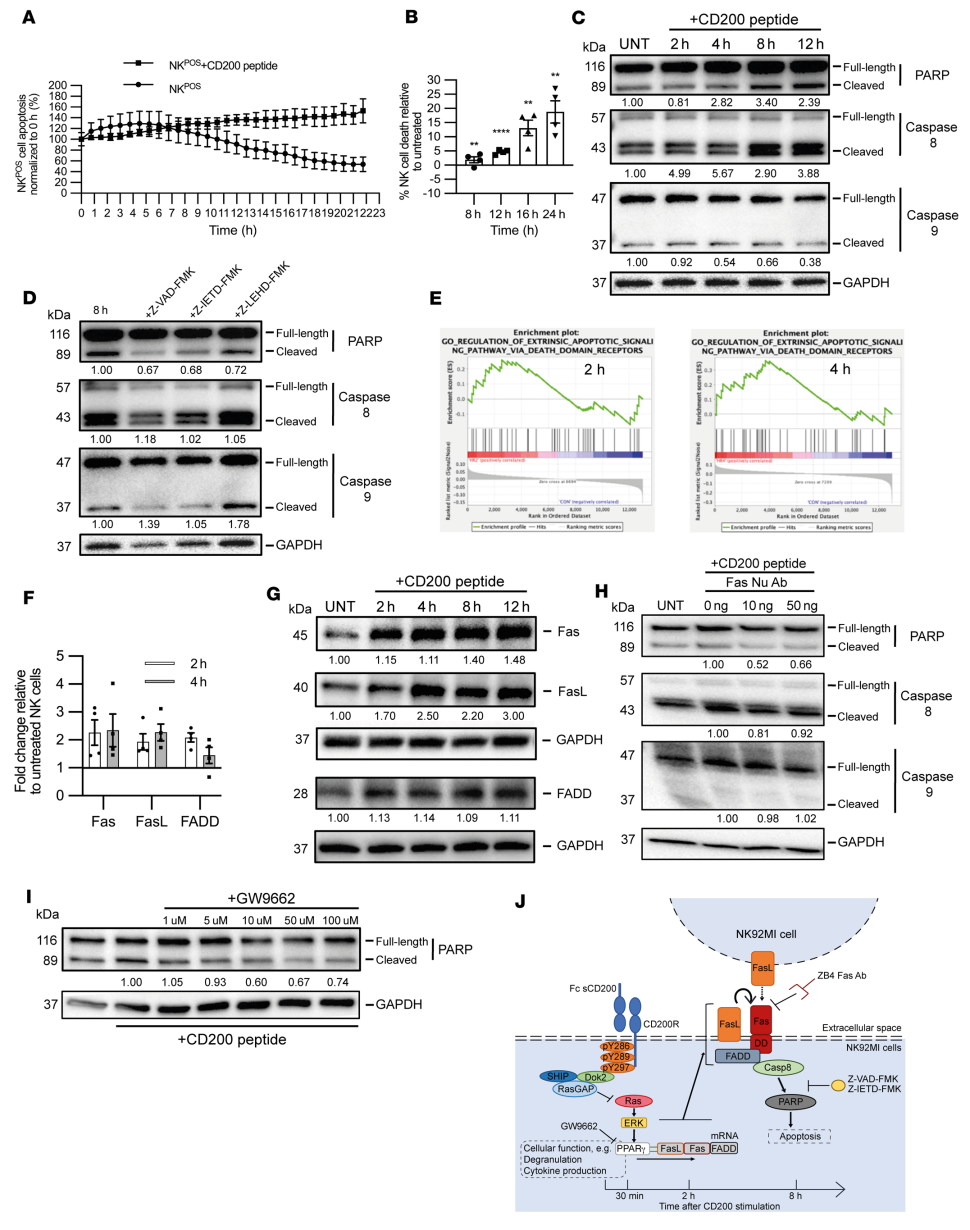
CD200 induced NK cell apoptosis. (**A**) Untreated (UNT) and CD200 peptide–treated NK^POS^ cells observed for apoptotic events by IncuCyte caspase 3 assay. (**B**) Apoptosis frequency between untreated and CD200 peptide–treated NK^POS^ cells was determined as a ratio of viability at 8, 12, 16, and 24 hours. (**C**) Immunoblots from untreated and CD200 peptide–treated NK^POS^ cells for various time points probed for PARP, caspase 8, caspase 9, and GAPDH. (**D**) Immunoblots for PARP, caspase 8, caspase 9, and GAPDH from untreated and CD200 peptide–treated (8 hours) NK^POS^ cells exposed to caspase inhibitors Z-VAD-FMK (pan), Z-IETD-FMK (caspase 8), and Z-LEDH-FMK (caspase 9). (**E**) Gene set enrichment plots obtained from differentially expressed genes from NK^POS^ cells incubated with CD200 peptide for 2 and 4 hours compared to untreated cells. (**F**) qPCR of NK^POS^ cells for Fas, FasL, and FADD genes after 2-hour or 4-hour CD200 peptide incubation relative to untreated. Expression was normalized to β-actin. Fold change was calculated relative to untreated NK cells according to the 2^–ΔΔCt^ method. (**G**) Immunoblots from untreated and CD200 peptide–treated NK^POS^ cells at various time points probed for Fas, FasL, FADD, and GAPDH. (**H**) Immunoblots for PARP, caspase 8, caspase 9, and GAPDH from untreated and CD200 peptide–treated (8 hours) NK^POS^ cells also exposed to anti-Fas monoclonal antibody (clone ZB4) at increasing concentrations. (**I**) Immunoblots for PARP and GAPDH from untreated and CD200 peptide–treated (8 hours) NK^POS^ cells also exposed to GW9662 at increasing concentrations. (**J**) Schematic summary of CD200-induced apoptosis. Western blot quantification is shown as a mean of 3 independent experiments. Data are presented as mean ± SD of 3 independent experiments. **P < 0.01; ****P < 0.0001 by 2-way ANOVA with Bonferroni’s post hoc test.

While using the Incucyte time-lapse caspase 3 coculture assay, we also observed NK^POS^ cell apoptosis during 24 hours of coculturing with HeLa^POS^ compared with HeLa^NEG^ cells (Supplemental Video 2). To determine whether CD200 could indeed sufficiently cause NK cell apoptosis to account for their absence within the BCC TME, NK^POS^ cells were incubated with CD200 peptide in an Incucyte caspase 3 assay (Figure 4A). We observed NK^POS^ cell apoptosis after 4 hours of incubation with the CD200 peptide in the caspase 3 assay, with a significant difference after 11 hours compared with untreated cells (*P* < 0.05; Figure 4A). When incubating NK^POS^ cells with and without CD200 peptide, there was a reduction in cell numbers relative to untreated cells after 4 hours that reached significance after 8 hours, resulting in 18.8% ± 3.93% NK^POS^ cell death at 24 hours (*P* < 0.01; Figure 4B). Cell protein lysates taken at various time points from NK^POS^ and NK^NEG^ cells treated with CD200 peptide were probed for poly(ADP-ribose) polymerase (PARP), as an early indicator of apoptosis. We observed a progressive increase in cleaved PARP levels after 4 hours with NK^POS^ cells (Figure 4C), similar in timing to the increase in cell death observed by the functional assays. However, there was no increase in PARP observed with NK^NEG^ cells (Supplemental Figure 4). These findings suggest that in addition to providing an inhibitory signal, CD200 also triggered NK cell apoptosis.

To define the mechanism for CD200-mediated NK cell apoptosis we performed a time-series Western blot analysis for cleaved caspase 8 (extrinsic apoptotic pathway) and caspase 9 (intrinsic apoptotic pathway) on lysates extracted from NK^POS^ and NK^NEG^ cells incubated with CD200 peptide (Figure 4C). We observed an increase in the cleavage of caspase 8 from as early as 2 hours, which continued throughout the time course (*P* < 0.01), but we observed no change in cleaved caspase 9. There was no increase in cleaved caspase 8 or 9 within NK^NEG^ cells (Supplemental Figure 4). As a positive control with fluoromethyl ketone–derivatized (FMK-derivatized) peptide Z-VAD-FMK, a cell membrane–permeant irreversible pan-caspase inhibitor without cytotoxicity, we observed reduced cleavage of PARP and caspase 8 (Figure 4D). Also, selective inhibition of caspase 8 with the Z-IETD-FMK inhibitor reduced the CD200-mediated cleavage of both PARP and caspase 8 within NK^POS^ cells. However, inhibition of caspase 9 with the Z-LEHD-FMK inhibitor did not reduce the cleavage of PARP or caspase 8. These results suggest that the caspase 8–mediated extrinsic pathway was involved in CD200-mediated NK^POS^ cell apoptosis.

As CD200-mediated apoptosis relied on the extrinsic pathway, with a time delay of 4 hours before apoptosis, we therefore hypothesized that apoptosis was mediated by a transcriptional event and thus we undertook transcriptomic analysis of 16,192 genes from untreated and 2- and 4-hour CD200 peptide–treated NK^POS^ cells (*n* = 3 replicates per condition). We chose 2- and 4-hour time points, even though apoptosis was evident later between 4 and 6 hours, to detect early transcriptional events and avoid the DNA damage response. Unbiased hierarchical clustering segregated the differentially expressed genes into 3 groups based on the duration of CD200 exposure (Supplemental Figure 5A). Gene Ontology apoptosis terms (http://geneontology.org/) were enriched at 2 hours (0043653: mitochondrial fragmentation involved in apoptosis process, 0042771: intrinsic apoptotic signaling pathway in response to DNA damage by p53 class mediator, and 0043523: positive regulation of apoptotic process; all *P* < 0.05) and 4 hours (1900118: negative regulation of execution phase of apoptosis, and 1900117: regulation of execution phase of apoptosis; both *P* < 0.01). At 4 hours, WikiPathways (https://www.wikipathways.org/index.php/WikiPathways) identified the term WP254: Apoptosis (*P* < 0.01) and Fas ligand (FasL) pathway and stress induction of heat shock protein (HSP) regulation as enriched (Supplemental Figure 5B). Gene set enrichment analysis (GSEA; Broad Institute, https://www.gsea-msigdb.org/gsea/index.jsp) identified the Gene Ontology term “regulation of extrinsic apoptosis signaling pathway via death domain receptors” (GO: 1902041) within both the 2- and 4-hour samples, with 39 of the 58 genes within the gene set shown to be enriched (enrichment score [ES] = 0.26, *P* = 0.374 and ES = 0.36, *P* = 0.063, respectively) (Figure 4E). Direct analysis of the 58 genes within the gene set GO: 1902041 demonstrated consistent increased expression of genes associated with the Fas apoptotic pathway (FasL, Fas, and FADD) at both 2- and 4-hour time points (Supplemental Figure 5C). This is consistent with the WikiPathways term WP254: Apoptosis and FasL pathway and stress induction of HSP regulation (Supplemental Figure 5B) and BioCarta (https://www.gsea-msigdb.org/gsea/msigdb/human/genesets.jsp?collection=CP:BIOCARTA) Fas pathway (Supplemental Figure 5D). However, only genes associated with the Fas death receptor signaling pathway members were concordantly enriched. Since these early time points may be responsible for the muted ESs, we sought to confirm enrichment of the FasL/Fas pathway by qPCR and immunoblotting. Upregulation of FasL, Fas, and FADD genes was observed after both 2- and 4-hour treatments when compared with untreated NK^POS^ cells (Figure 4, F and G). As FasL can be membrane bound or secreted, we undertook an ELISA of culture supernatants from NK^POS^ cells treated with CD200 peptide. There was no discernible increase in soluble FasL within the cell culture supernatant (Supplemental Figure 5E), suggesting that NK cell apoptosis predominated through cell-membrane-bound cell-cell interactions (fratricide). Blocking FasL-Fas interactions with an anti-Fas monoclonal antibody (clone ZB4) by addition to culture of NK^POS^ cells treated with CD200 peptide prevented cleavage of PARP and caspase 8 (Figure 4H). Hence, CD200 signaling–induced apoptosis of the NK^POS^ cells was mediated by overexpression of Fas death receptor pathway members.

Intriguingly, WikiPathways analysis of our data set also identified WP2456: HIF1A and PPARG regulation of glycolysis and WP1946: Cori cycle gene set enrichment (*P* < 0.01) (Supplemental Figure 5B). We also observed enrichment of the KEGG (https://www.genome.jp/kegg/pathway.html) PPAR signaling pathway (ES = 0.46, *P* < 0.05; Supplemental Figure 5F). When assessing the expression of experimentally verified PPAR target genes from the 3 PPAR isoforms (52), we found that the PPARγ target genes demonstrated concordant gene expression in our data set (Supplemental Figure 5G). Moreover, FasL-mediated apoptosis regulated by PPARγ has previously been described (53). Therefore, as the MAPK pathway negatively regulates PPARγ-regulated transcription, we next hypothesized that CD200 signal transduction led to an increase in PPARγ-regulated gene transcription, including FasL gene expression (53). To determine whether CD200 signaling induced within the human NK^POS^ cell line has human physiological relevance, we next sought to determine whether primary NK cells subject to CD200 signaling would also undergo apoptosis. NK cells enriched from human PBMC isolates were treated with human CD200 peptide for 4 hours and then the CD45^+^CD3^–^CD56^+^CD200R^+^ and CD45^+^CD3^–^CD56^+^CD200R^–^ fractions were used for gene expression analysis or labeled with annexin V to determine apoptosis frequency (Supplemental Figure 6A). CD200 signaling in freshly isolated primary CD200R^+^ NK cells led to increased expression of apoptotic genes expressing Fas, FasL, and FADD (Supplemental Figure 6B) and resulted in an increased rate of apoptosis when compared with CD200R^–^ NK cells (*P* < 0.05; Supplemental Figure 6C). GW9662 is a potent irreversible antagonist of PPARγ; therefore, we treated NK^POS^ cells with CD200 peptide, ERK inhibitor, and GW9662 for 8 hours. As expected, CD200 peptide and ERK inhibition led to an increase in Fas, FasL, and FADD, whereas GW9662 treatment dramatically reduced Fas and FADD expression (Supplemental Figure 6D). Consistent with this, we observed a decrease in the levels of cleaved PARP induced by CD200 following GW9662 treatment (Figure 4I). Furthermore, we observed greater NK cell apoptosis and loss of CD200R-positive cells in CD200-expressing xenografts (Figure 3F and Supplemental Figure 3). In summary, CD200 signal transduction in NK cells reduced MAPK signaling that in turn facilitated PPARγ gene transcription of the Fas death receptor family members, leading to time-dependent autoregulatory activation–induced NK cell apoptosis (Figure 4J).

### Matrix metalloproteinases contribute to ectodomain shedding of sCD200.

**Figure 5:**
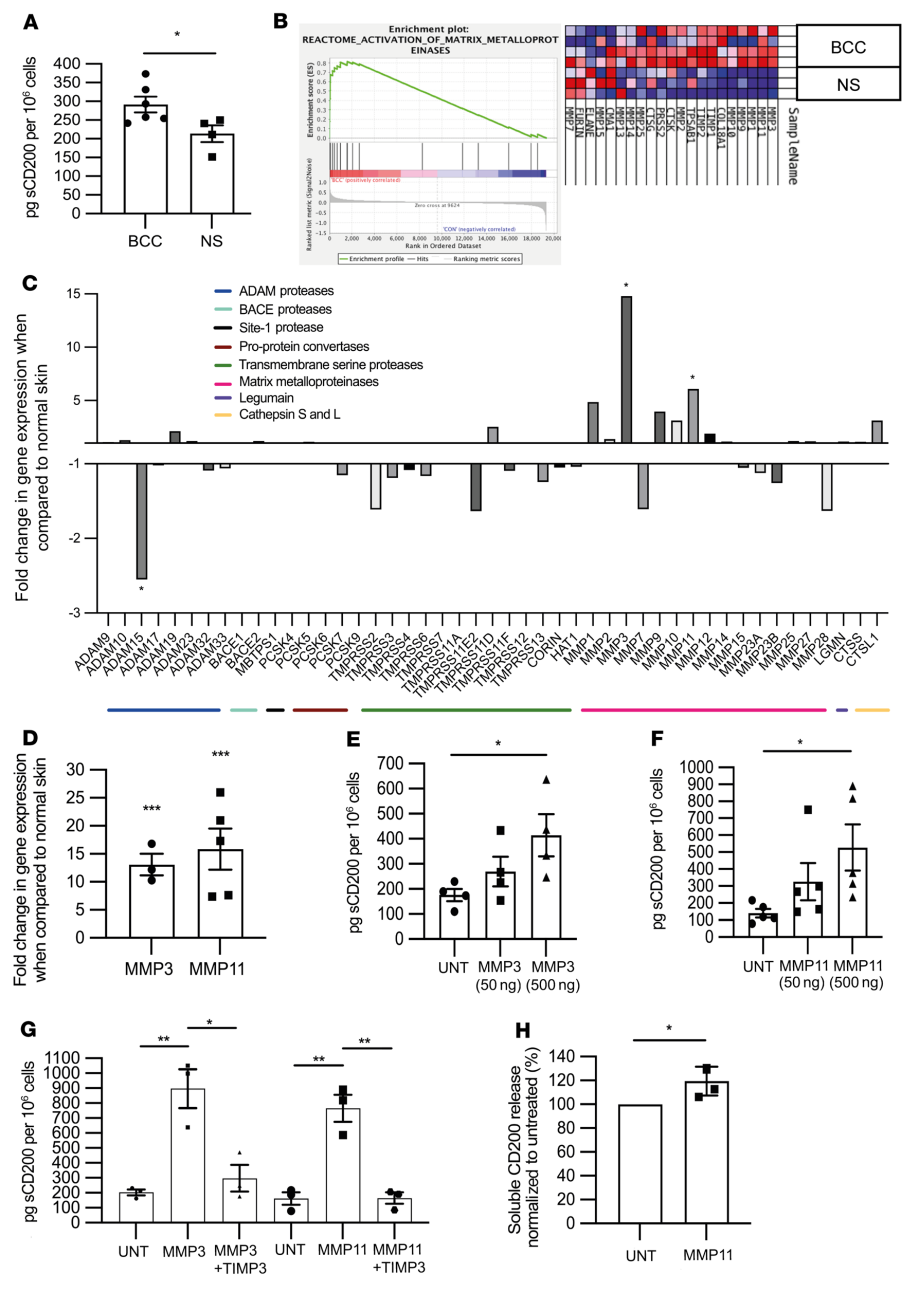
Matrix metalloproteinases contribute to CD200 ectodomain shedding. (**A**) ELISA determination of active sCD200 per 1 × 10^6^ cells from culture supernatant of primary human BCC and primary human normal skin after 24 hours. (**B** and **C**) Transcriptomic analysis of BCC and normal skin, “activation of matrix metalloproteinases” gene set enrichment (**B**), and relative expression of putative proteases (**C**). (**D**) qPCR-determined relative MMP3 and -11 gene expression from primary human BCC and normal skin tissue samples normalized to β-actin. (**E**–**G**) ELISA determination of active sCD200 pg per 1 × 10^6^ cells from culture supernatant of HeLA^POS^ cells either untreated (UNT) or with addition of MMP3 (**E**) or MMP11 (**F**) for 24 hours, and together with TIMP3 (**G**). (**H**) ELISA determination of relative active sCD200 per 1 × 10^6^ cells from culture supernatant of primary human BCC after treatment with 500 ng of MMP11 for 24 hours. Data are presented as mean ± SD of 3 independent experiments. *P < 0.05; **P < 0.01; ***P < 0.001 by 2-tailed Student’s *t* test.

CD200 mRNA and protein levels were greater in BCC compared with SCC (Supplemental Figure 7, A and B). Similarly, transcript levels in BCC were greater than in normal skin, consistent with a greater BCC CD200-expressing population (Supplemental Figure 7C). Although CD200 expression in BCC is limited to a small cancer stem cell population, we observed higher levels by both qPCR and Western blotting. We next sought to determine whether CD200 in BCC could be released into the TME as soluble CD200 (sCD200). Using a CD200 ELISA to detect biologically active sCD200 in culture supernatant, we determined that primary human BCC exhibited significantly higher levels of sCD200 (291.9 ± 21.10 pg/10^6^ cells [*n* = 6]) than primary human normal skin (213.50 ± 22.31 pg/10^6^ cells [*n* = 4]; *P* < 0.05; Figure 5A). Hence, BCC cells express CD200 and appear to release sCD200 into the TME.

To identify proteases that could cleave and release sCD200 into the BCC TME, we undertook transcriptomic analysis of BCC (*n* = 4), SCC (*n* = 3), and normal skin (*n* = 3). We identified 1,423 and 1,663 differentially expressed genes between BCC and normal skin and between BCC and SCC, respectively (adjusted *P* < 0.05). In keeping with the prominent stroma around BCC, a volcano plot (fold change > 2.0 or < –2.0, adjusted *P* < 0.01) identified 16 of the 24 overexpressed genes in BCC compared with normal skin that were associated with extracellular matrix remodeling: collagen genes (COL1A1, -1A2, -1A2, -3A1, -5A1, -5A2, and -6A3), proteoglycans and glycoproteins (VCAN, FBN3, TNC, CSPG4, and LUM), other extracellular proteins (SPON2 and CALD1), and proteases (MMP11) (Supplemental Table 1). Unsupervised hierarchical clustering defined 235 differentially expressed genes (adjusted *P* < 0.05; Supplemental Figure 7D), from which there was enrichment of the Reactome (https://reactome.org/) gene set “activation of matrix metalloproteinases” in BCC compared with normal skin (ES = 0.81; Figure 5B). Enrichment was observed for metallocarboxypeptidase (GO: 0004181; ES = 0.63, *P* = 0.08), metalloendopeptidase (GO: 0004222; ES = 0.62, *P* = 0.16), metalloexopeptidase (GO: 0008235; ES = 0.52, *P* = 0.14), and metallopeptidase (GO: 0008237; ES = 0.51, *P* = 0.14) activity in BCC compared with normal skin (Supplemental Figure 7E). Gene expression analysis of individual BCC proteases relative to normal skin and SCC identified MMP3 and -11 as potential candidates for CD200 sheddases (Figure 5C and Supplemental Figure 7F, respectively). Only MMP11 was significantly elevated in BCC compared with SCC (*P* < 0.01; Supplemental Figure 7F). Likewise, substrates for MMP3 and -11, but not ADAMs proteases, were increased in BCC compared with normal skin (Supplemental Figure 7G). Hallmark gene set enrichment also included MMP11 and -3 as genes involved in epithelial mesenchymal transition, with enrichment in BCC versus normal skin (ES = 0.77, *P* < 0.05) and BCC versus SCC (ES = 0.66, *P* = NS) (data not shown). qPCR of BCC and normal skin tissues confirmed the increase in MMP3 (13.1-fold, *P* < 0.001) and MMP11 (15.9-fold, *P* < 0.001) (Figure 5D). There was no significant increase in the cell-surface-bound proteases, ADAMs, in BCC determined by microarray and qPCR analyses (Supplemental Figure 7, H and I).

We next determined whether MMP3 and MMP11 could act as CD200 sheddases. Addition of MMP3 and MMP11 to CD200^+^ HeLa cells in culture showed a concentration-dependent increase in sCD200 levels in the supernatant (Figure 5, E and F). When compared with unstimulated cells, both MMP3 and 11 were shown to increase the levels of sCD200 in the supernatant at both 50 ng (269.80 ± 59.24 vs. 175.80 ± 24.87 pg/10^6^ cells and 325.70 ± 109.70 vs. 140.60 ± 24.89 pg/10^6^ cells, respectively) and 500 ng (414.30 ± 83.95 vs. 175.80 ± 24.87 pg/10^6^ cells and 527.20 ± 135.80 vs. 140.60 ± 24.89 pg/10^6^ cells, respectively) (*P* < 0.05; Figure 5, E and F). Coincubation with the tissue inhibitor of metalloproteinases 3 (TIMP3) reversed sCD200 levels induced by both MMP3 (*P* < 0.05) and MMP11 (*P* < 0.01) (Figure 5G). TIMP3 did not affect NK^POS^ cell viability or function (data not shown). In primary human BCC culture, we found that MMP11, but not MMP3 (data not shown), had the potential to increase sCD200 levels in the supernatant when compared with the untreated control (*P* < 0.05; Figure 5H). Hence, MMP11 expression in BCC is responsible for ectodomain shedding of biologically active sCD200 from cancer stem cells into the surrounding TME.

### The CD200 TME targets NK cells.

**Figure 6:**
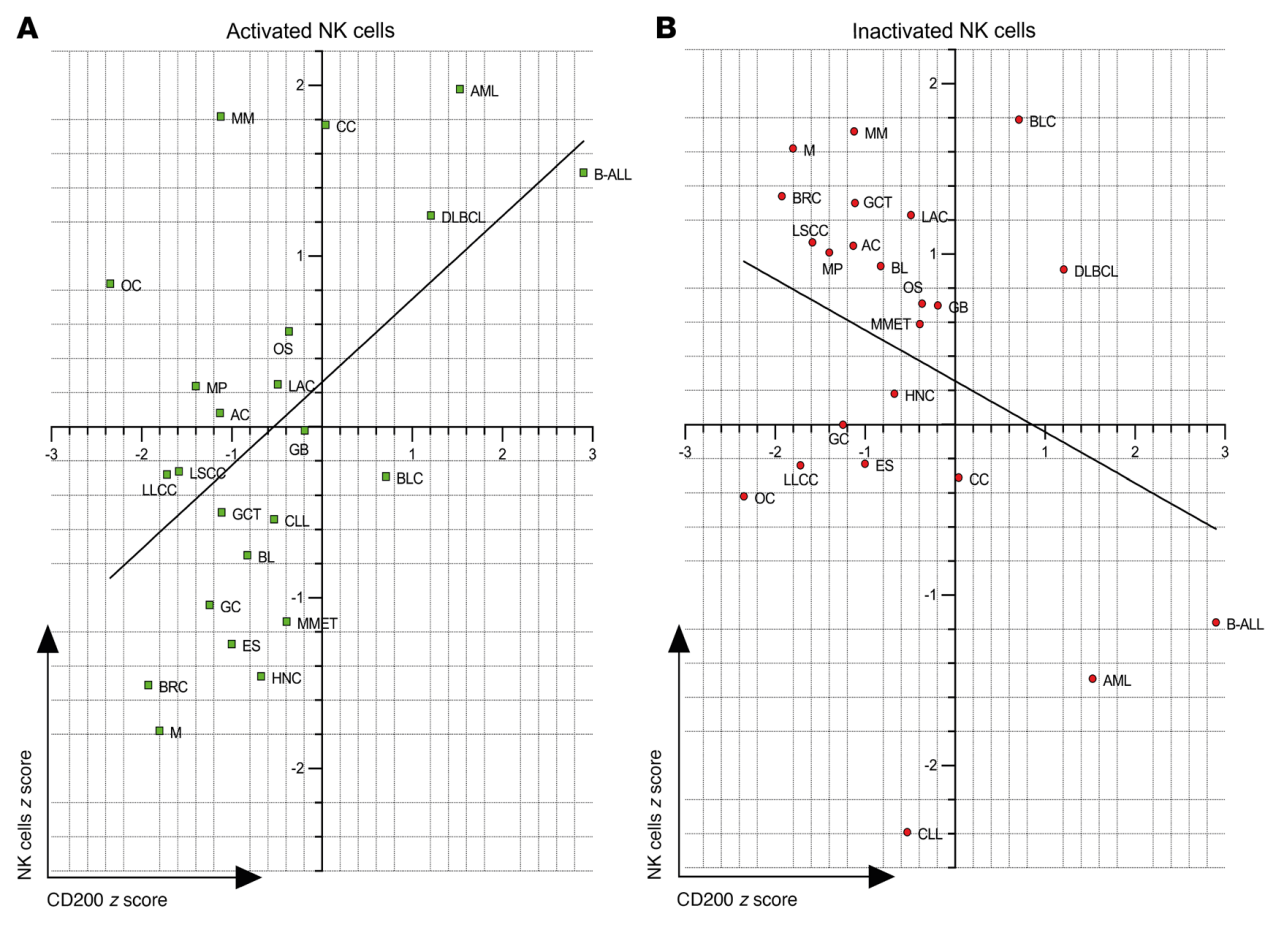
Cancer outcomes associated with CD200 expression and NK cell activation. (**A** and **B**) Concordance and differences in prognostic associations between CD200 expression and activated (**A**) or inactivated (**B**) NK cells in acute myeloid leukemia (AML), astrocytoma (AC), B cell acute lymphocytic leukemia (B-ALL), breast cancer (BRC), Burkitts lymphoma (BL), chronic lymphocytic leukemia (CLL), colon cancer (CC), diffuse large B cell lymphoma DLBCL, lung adenocarcinoma (LAC), Ewings sarcoma (ES), gastric carcinoma (GC), glioblastoma (GB), head and neck carcinoma (HNC), lung large cell carcinoma (LLCC), lung small cell carcinoma (LSCC), germ cell tumor (GCT), metastatic melanoma (MMET), melanoma primary (MP), multiple myeloma (MM), osteosarcoma (OS), meningioma (M), and ovarian cancer (OC). The *r*^2^ values for activated NK cells and inactivated NK cells were 0.28 and 0.12, respectively, as determined using linear regression analysis.

Many different cancers exhibit diminished NK cell numbers within the TME, while their presence often denotes good prognosis, and their infusion can be therapeutic (54, 55). To determine whether CD200-mediated dysregulation and apoptosis of NK cells could influence outcomes in many cancers, we analyzed the prediction of clinical outcomes from genomic profiles data set (PRECOG, http://precog.stanford.edu). Recently, this data set was analyzed by cell type identification by estimating relative subsets of known RNA transcripts (CIBERSORT) to assign outcome *z* scores for immune cell profiles across 25 cancer types (56). Using the iPRECOG database (https://precog.stanford.edu), we determined outcome *z* scores based on CD200 expression and determined a direct correlation between CD200-based outcomes and “activated” NK cell outcomes (*r*^2^ = 0.2783, *P* < 0.01; Figure 6A) and also a weak inverse correlation with “rested” NK cell outcomes (*r*^2^ = 0.1156, *P* = 0.1124; Figure 6B). Our analysis of CD200 expression *z* scores was startling in that 9 of the 31 solid cancers analyzed had unfavorable outcomes, notably with worse outcomes for solid cancers that are associated with abnormal or absent NK cell immune responses, including head and neck, esophageal, bladder, and liver cancers (Supplemental Table 2). Furthermore, other immune cell phenotypes did not correlate with CD200 *z* scores (Supplemental Figure 8, A–R, and Supplemental Table 3), suggesting that outcomes associated with CD200 expression were predominantly mediated via activated NK cells.

**Figure 7:**
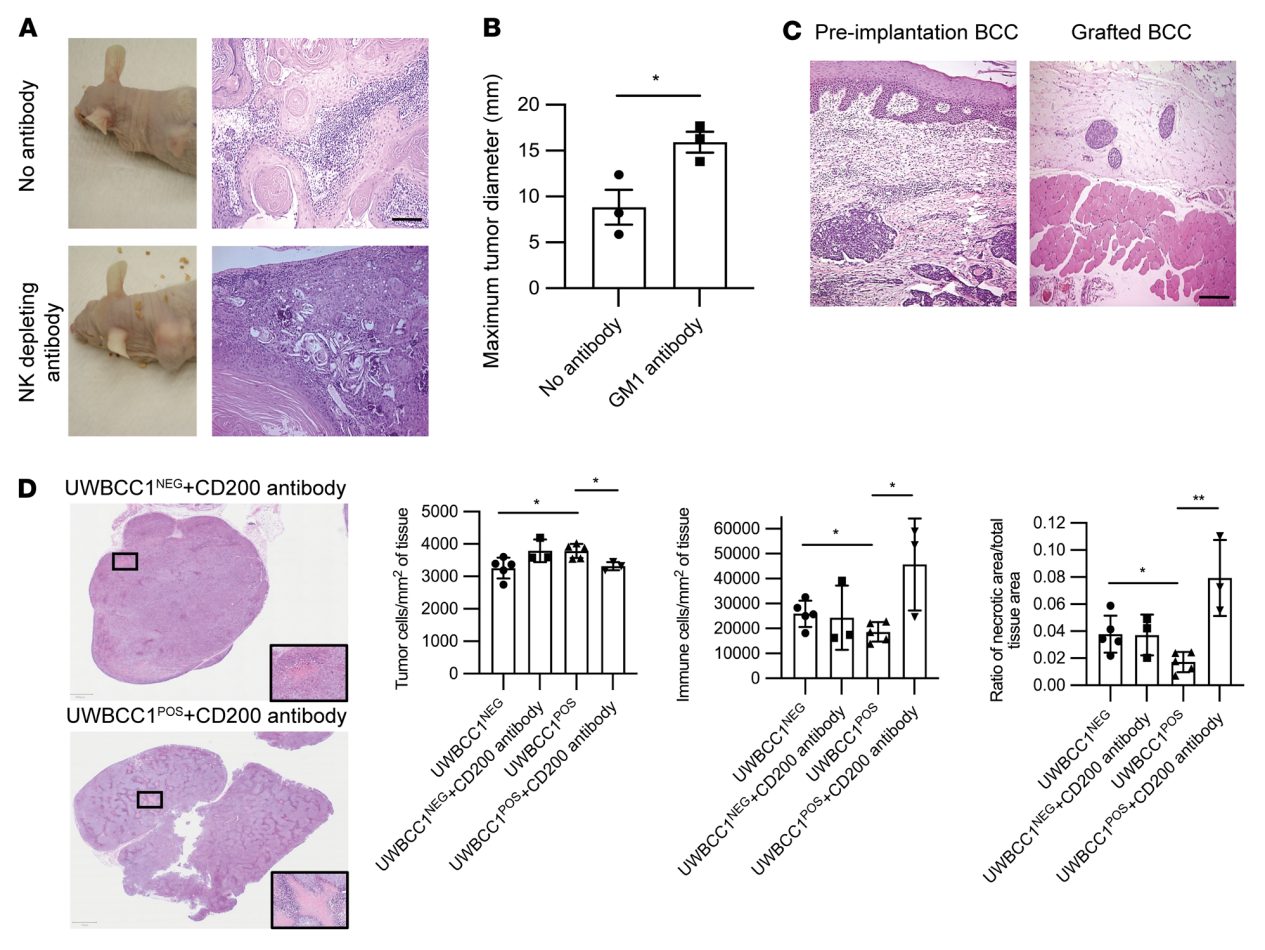
NK depletion facilitates skin cancer growth. (**A**–**C**) Dissociated primary human SCC (**A** and **B**) and BCC (**C**) cells were grafted into the subcutaneous tissues of nude mice treated without and with intraperitoneal injection of NK cell–depleting antibody anti–asialo GM1 (50 μL) 1 day prior to tumor graft. (**A** and **B**) SCC growth was enhanced in the NK-depleting antibody cohort. (**C**) BCC growth only occurred with NK-depleting antibody pretreatment. Scale bars: 100 μm. (**D**) Day 7 tumors from nude mice were grafted with 1 × 10**^6^** UWBCC1^POS^ or UWBCC1^NEG^ cells (*n* = 5 each) treated daily with intraperitoneal administration of anti-CD200 antibody: histological analysis of tumor cellularity, inflammatory cell infiltration, and necrosis. Data are presented as mean ± SD of 3 independent experiments. *P < 0.05; **P < 0.01 by 2-tailed Student’s *t* test.

To test whether the establishment of a CD200 TME that specifically targets NK cells was essential for BCC growth, we pretreated nude mice with an NK cell–depleting antibody before grafting primary human BCC and SCC cells. A temporary NK cell–depleting anti–asialo GM1 antibody was administered by intraperitoneal injection the day before tumor grafting, as previously reported (57). CD200 expression was determined by qPCR before grafting (Supplemental Figure 9A). SCC grafted cells grew irrespective of anti–asialo GM1 antibody administration. However, mice treated with anti–asialo GM1 antibody had significantly larger SCC tumors (15.93 ± 1.14 mm [*n* = 3]) compared with untreated tumors 8.83 ± 1.90 mm [*n* = 3]; *P* < 0.05) (Figure 7, A and B). In contrast, BCC grafts did not establish growth in untreated mice, consistent with the susceptibility to NK cell killing before establishment of a CD200 TME. Pretreatment with NK-depleting anti–asialo GM1 antibody enabled BCC growth to occur and thereafter be maintained (*n* = 3 different primary BCCs; Figure 7C). The growth characteristics of BCC xenografts after NK-depleting antibody was similar to that observed previously with etoposide pretreatment (58, 59). The development of tumor heterogeneity prevented CD200^+^ cell detection by immunohistochemistry in the BCC and SCC xenografts (Supplemental Figure 9B). Next, we sought to determine whether the level of CD200 expression within the BCC TME influenced NK cell cytotoxicity. Since NK cell cytotoxicity is dependent on expression of activation receptors, we examined NCR1–3 and KLRK1 expression in a microarray data set of 21 BCCs (60). The BCC samples were defined based on CD200 expression, by determining the upper (CD200 high) and lower (CD200 low) quartiles, which yielded a 2-fold difference in CD200 expression (Supplemental Figure 9C). Acknowledging the low sample numbers, we found that CD200-high BCC tissue samples had lower expression of the NK activation markers NCR3 (NKp30, *P* = 0.18), NCR2 (NKp44, *P* = 0.02), and NCR1 (NKp46, *P* = 0.04). Noteworthily, comparing resistant (*n* = 9) and sensitive (*n* = 4) BCC samples similarly reflected increased CD200 expression and lower expression of NK cell receptors (Supplemental Figure 9D). Finally, we sought to determine whether administration of a CD200-blocking antibody would be sufficient to restore NK cytotoxicity in vivo. UWBCC1^POS^ and UWBCC1^NEG^ cells were grafted into the flank of mice and allowed to establish tumor growth for 4 weeks, when tumors were approximately 10 mm in diameter, and intraperitoneal CD200-blocking antibody was administered daily for 7 days. A further 7 days after treatment, larger UWBCC1^POS^ tumors after CD200-blocking antibody treatment demonstrated reduced cellularity (*P* < 0.05) from increased immune cell infiltrate (*P* < 0.05) and tumor necrosis (*P* < 0.05) (Figure 7D). In summary, BCC cells are susceptible to NK cell killing, but are protected once they establish a CD200 TME that precludes NK cell infiltration by inducing NK cell dysfunction and apoptosis. Hence, the CD200 microenvironment appears to be essential for BCC growth.

## Discussion

In the face of a competent immune system, tumor tissues with mutant protein expression undergo remodeling to escape both innate and adaptive immune detection. Tumor cells escape immune recognition by (a) expression of immune modulatory proteins, (b) selection of less immunogenic clones, or (c) through the induction and recruitment of immunosuppressive immune cells within the TME (61). Clonal selection is evident in many cancers, wherein certain clones demonstrate preferential expansion (62). In the case of hierarchical cancer growth it is essential that specifically cancer stem cells evade immune detection, which in the case of BCC may account for the slow tumor growth with reduced metastatic potential.

NK cell activation–induced cell death represents an important mechanism for both homeostatic regulation of NK cell numbers and peripheral tissue tolerance. Fas and FasL activation–induced cell death has been reported as a mechanism for peripheral T cell tolerance (63). Yet NK cell regulation has only been regarded as a balance between stimulatory and inhibitory signals determining NK cell activation, without consideration of NK cell numbers beyond recruitment (64–66). Among the potential regulatory mechanisms, soluble HLA class I molecules have also been shown to induce autoregulatory FasL- and Fas-mediated NK cell apoptosis (67). Poggi et al. reported up to 80% Fas-induced time-dependent apoptosis of primary blood–derived NK cells when cocultured with a variety of cancer cell lines for 48 hours (68). Hence, deregulation of activation-induced NK cell death may account for the absence of NK cells within the antitumor immune infiltrate observed in many cancers, including BCC.

CD200 is a highly conserved type 1 membrane glycoprotein that is expressed primarily by the brain, smooth muscle, cardiomyocytes, neural cells, placenta, testis, and human hair follicle bulge keratinocyte stem cells (69, 70). CD200 is expressed in a number of malignancies and has been associated with poor outcome, including melanoma, acute myeloid leukemia, multiple myeloma, chronic lymphocytic leukemia, renal carcinoma, bladder cancer, ovarian carcinoma, and colon carcinoma (28, 71–75). Yet for many cancer types CD200 expression is limited to a subset of tumor cells, notably BCC where its expression is restricted to a small cancer stem cell population (76). CD200 is also liable to ectodomain shedding by sheddases via a process that similarly releases many functionally active cytokines, chemokines, cytokine receptors, and other immunoregulatory molecules (77). The extracellular portion of CD200 once cleaved is released into the extracellular matrix as bioactive sCD200 (78). Circulating levels of sCD200 have also been found to correlate with disease severity in a number of inflammatory diseases, such as systemic lupus erythematosus, endometriosis, and bullous pemphigoid (49, 79, 80). ADAM28 has previously been shown to be responsible for sCD200 release in chronic lymphocytic leukemia (78). In hematological malignancies where sCD200 is shed directly into the circulation, sCD200 levels directly correlate with disease stage and patient prognosis (81). Circulating sCD200 has also been observed in patients with glioblastoma multiforme, with the highest levels in those patients demonstrating tumor progression despite treatment (82). Hence, tumors that release sCD200 can influence the antitumor immune response throughout the TME.

Activation of the cognate CD200R on lymphocytes, myeloid, and NK cells leads to suppression of the immune response via recruitment of inhibitory effectors such as RasGAP, SHP, and Csk that reduce intracellular MAPK signaling (83–86). Our findings from analysis of the PRECOG and iPRECOG databases has found that cancer patient adverse outcomes related to CD200 expression levels are directly tied to NK cell activation. As mediators of MAPK signaling, ERKs phosphorylate PPARγ on Ser82 and Ser112, leading to ubiquitination and sumoylation of PPARγ to block transactivation (87). In addition, MEK1 causes nuclear export of PPARγ, where in the cytoplasm it is targeted for proteasomal degradation (88). Hence, CD200 signaling relinquishes PPARγ to transactivate gene expression, which includes FasL, Fas, and FADD genes (53). The PPARγ agonists, thiazolidinediones, are known to induce apoptosis in a number of different cell lines, including cancer cell lines (89–93). Intriguingly, use of pioglitazone, an antidiabetes glitazone, has also been associated with a dose-dependent increased incidence of bladder cancer, leading to an FDA label warning (94). Furthermore, there is an increase in prostate cancer with over 9.5 years of pioglitazone use; however, the association has not reached statistical significance (95). Similarly, endogenous, weak PPARγ agonists such as fatty acids and eicosanoids have long been associated with an increased incidence of cancer, and their effect may be circumvented by COX2 inhibitors (96). In contrast to COX2 inhibitors, a select group of nonsteroidal antiinflammatory drugs, including ibuprofen, are PPARγ agonists and may account for the difference in cancer incidence with their prolonged use in susceptible tissues (97, 98). Here we have shown that inhibition of PPARγ transactivation alone is sufficient to block CD200-induced NK cell apoptosis.

In addition to T lymphocytes, early experimental tumor models also demonstrated increased cancer burden and metastasis in mice with NK cell deficiencies, suggesting that NK cells may also be important in tumor immune evasion (32, 99–102). Abnormal or absent NK cell immune responses have now been observed in a number of cancers supported by improved TME immune cell profiling techniques, including acute myeloid leukemia and bladder, liver, head and neck, and lung cancers (103–109). Moreover, the absence of NK cell signatures has been linked to poor-prognosis *z* scores in an analysis of the Stanford PRECOG database of 30,000 transcriptomes from 166 cancers, encompassing 39 distinct malignancies, using CIBERSORT-defined tumor-associated immune cell profiles (56). Many cancer types therefore demonstrate an abnormal or absent NK cell immune response.

The contribution of the TME in immune evasion represents an emerging hallmark of cancer (110). The importance of the TME in sustaining BCC growth has intrigued researchers for many decades. Experiments conducted in the early 1960s showed that autotransplanted human BCC tumor tissue growth required concurrent transplantation of the TME (111). The BCC TME is supported by platelet-derived growth factor as a transcriptional product of hedgehog-signaling-driven tumor growth (112). Transcriptional profiling of BCC herein identified many genes involved in tissue remodeling, including collagens, proteoglycans, and metalloproteinases. But ultimately it may be the immunological barrier created by sCD200 in the TME that is fundamental for BCC growth. In the absence of an intact TME BCC cell engraftment in immunosuppressed mice, which still possess NK cells, is dependent on prior NK cell depletion with anti–asialo GM1 antibody (described herein), etoposide, or splenectomy and antilymphocyte serum (113, 114). These findings suggest that an established BCC TME is responsible for NK cell immune evasion. Although CD200 is expressed by less than 4% of BCC cells, which exhibit a cancer stem cell phenotype, MMP11 facilitates high levels of sCD200 secretion into the TME. Our findings suggest that sCD200 in the BCC TME leads to activation-induced NK cell death, resulting in a near absence of NK cells within the tumor infiltrate, and this mechanism may readily apply to other cancer types.

## Methods

Further information can be found in Supplemental Methods.

### Experimental models

#### Mouse xenografting.

Since they still have a robust NK cell response, NU(NCr)-*Foxn1^nu^* mice (6- to 8-week-old females; Charles River) were used to study the impact of CD200 expression on in vivo tumor growth. Mice were anesthetized with isoflurane and 1 × 10^6^ CD200^+^ (*n* = 5) and CD200^–^ (*n* = 5) HeLa cells were injected into the flank and allowed to establish over 5 days, after which mice were culled and harvested. PBMCs were extracted from the blood for flow cytometry and tumors were fixed and embedded in paraffin for assessing CD45^+^ and NK1.1^+^ immune cell infiltration into the TME.

#### Tumor cell lines.

The cervical adenocarcinoma cell line HeLa (CCL-2) and the NK cell line NK92-MI (CRL-2408) were obtained from the American Type Culture Collection (ATCC). The IL-2–dependent NK cell line (NKL) was provided by Michael Robertson (School of Medicine, Indiana University, Bloomington, Indiana, USA) (115). The BCC cell line (UWBCC1) was provided by Vladimir Spiegelman (Pennsylvania State University, Hershey, Pennsylvania, USA) (116). HeLa cell lines were maintained in a T75 flask with RPMI 1640 plus L-glutamine, 10% FBS, and 1% penicillin-streptomycin (P-S) at 37°C in a 5% CO_2_ incubator. The NK92MI cell line was maintained in a T150 flask with RPMI 1640 plus L-glutamine, 10% heat-inactivated FBS, 10% heat-inactivated horse serum, and 1% P-S at 37°C in 5% CO_2_. The IL-2–dependent NKL cell line was maintained in a T75 flask with RPMI 1640 plus 10% FBS, IL-2 (1000 U/mL), and 1% P-S at 37°C in 5% CO_2_.

### Methods details

#### Generation of CD200^+^ and CD200^–^ HeLa cell lines.

Complementary DNA for CD200 was provided by the I.M.A.G.E. Consortium (clone ID 5299899), and subsequently subcloned into the PINCO retroviral expression vector, which coexpresses GFP from an internal CMV promoter (constructed in-house using an expression vector gifted to Alex Tonks by Pier Pelicci, European Institute of Oncology, Milan, Italy (117). The expression levels of CD200 were confirmed in both the CD200^+^ and CD200^–^ HeLa cell lines through flow cytometry.

#### HeLa and NK cell coincubation.

HeLa cells were detached using Versene (Gibco) and seeded (20,000 cells/well) into a white-walled 96-well plate and allowed to adhere overnight. The following day, cells were detached and counted to determine the number of cells in each well. NK92MI cells were added to the HeLa cells at different E:T ratios (described in figure legends). Coincubations were left for 4 hours, after which the suspended NK cells and the supernatant were removed and the remaining tumor cells at the bottom of the well were washed thoroughly with PBS. Tumor cell death was measured relative to untreated tumor cell wells using the CellTiter Glo assay as described in the Supplemental Methods.

#### CD200 peptide treatment of NK92MI cells.

NK92MI cells were plated in either a 96- or 24-well plate. A CD200-Fc chimeric protein (R&D Systems), containing Gln31–Gly232 of CD200 (QVQVVTQDEREQLYTPASLKCSLQNAQEALIVTWQKKKAVSPENMVTFSENHGVVIQPAYKDKINITQLGLQNSTITFWNITLEDEGCYMCLFNTFGFGKISGTACLTVYVQPIVSLHYKFSEDHLNITCSATARPAPMVFWKVPRSGIENSTVTLSHPNGTTSVTSILHIKDPKNQVGKEVICQVLHLGTVTDFKQTVNKG) that includes the ectodomain, was added to the cells at a concentration of 4 μg/10^6^ cells for indicated time points, after which cell viability was determined and/or protein and RNA was extracted. Various inhibitors were added to the cocultures as described throughout.

#### Cell-conditioned media of cocultures.

HeLa cells were seeded in a 96-well plate and coincubated with 200 μL of either an effector cell line (NK92MI) or with CD200 peptide as described above. Cells were coincubated for the indicated periods of time, after which the suspended NK92MI cells and the conditioned media/supernatant were transferred to a 96-well round-bottom plate. The plate was centrifuged at 200*g* for 5 minutes to pellet the NK92MI cells at the bottom of the well and supernatant removed.

#### Immunofluorescence.

Immunofluorescence was performed on either frozen OCT-embedded or paraffin-embedded sections (28). Primary and secondary antibodies used can be found in Supplemental Table 4.

#### Immunohistochemistry.

Immunohistochemistry was performed on paraffin-embedded sections as previously described (28). Primary and secondary antibodies used can be found in Supplemental Table 4.

#### Flow cytometry.

HeLa cells in culture were detached using Versene. Cells were washed with FACS buffer (0.05% sodium azide and 0.5% BSA in PBS) before primary antibody staining (Supplemental Table 4). All antibody incubations were carried out for 30 minutes at 4°C. Unbound antibodies were removed by washing with FACS buffer twice by centrifugation. All centrifugations were performed at 250*g* for 5 minutes at 4°C. Samples were gated on the basis of forward and side scatter. Doublets and dead cells were excluded. Single-stained samples were used as compensation controls and an isotype control antibody was used to determine background fluorescence. Data were processed using FlowJo analysis software (FlowJo, LLC).

### Microarray of primary human tissue samples and the NK92MI cell line

RNA for microarray was extracted and RNA quality was assessed as described in Supplemental Methods. RNA was amplified and cDNA was prepared using the Illumina TotalPrep RNA Amplification Kit, which was used to generate biotinylated amplified RNA for hybridization with Illumina Sentrix arrays. Samples were applied to the Illumina HumanHT-12 v4 Expression BeadChip, which provided genome-wide transcriptional coverage of well-characterized human genes. Nine NK cell samples (3 untreated NK, 3 with 2-hour treatment, and 3 with 4-hour treatment with CD200 peptide; accession number E-MTAB-12035) and 10 tissue samples (4 BCC, 3 SCC, and 3 normal skin; accession number E-MTAB-12034) were applied to the chip using the Direct Hybridization assay protocol. A full protocol and reagent list can be found on the Illumina website.

### Statistics

Statistical tests are described in the figure legends and were performed using GraphPad Prism v8. A *P* value of less than 0.05 was considered significant. NS, nonsignificant; *P < 0.05; **P < 0.01; ***P < 0.001; ****P < 0.0001. For each experiment, *n* represents the number of experimental replicates.

### Study approval

#### Animals.

All animal experiments carried out in this study were performed in accordance with a UK Home Office Licence (Project License 30/3382).

#### Patient samples.

BCC, SCC, normal skin, and blood samples were obtained after a UK NHS R&D and Local Research Ethics Committee study approval (protocol number 09-WSE-02-1). Patients were recruited from Hywel Dda and Cardiff and Vale University Health Boards after informed written consent.

## Author contributions

All authors conceived and designed the experiments. HJM, ER, SL, AG, and BYS conducted in vitro and in vivo biological experiments and analysis. HJM, ER, SL, and GED conducted ELISA experiments. GKP designed research. AT, RD, and ECYW contributed new reagents/analytic tools. SL, ER, GT, and HJM performed immune cell flow cytometry and subsequent analysis. CO assisted in biological studies, data analysis, and data interpretation. HJM, ER, and AG performed microarray and subsequently analyzed the data. ER, SL, HJM, and CL helped in Western blot experiments. JG and KAP performed immunofluorescence on primary human tissue. GKP and HJM wrote the manuscript, with input from all the other authors.

## Supplementary Material

Supplemental data

Supplemental video 1

Supplemental video 2

## Version 1 09/08/2022

In-Press Preview

## Version 2 11/01/2022

Electronic publication
