# Supplementary material for: CD200 ectodomain shedding into the tumor microenvironment leads to NK cell dysfunction and apoptosis
Source: J Clin Invest. 2022 Nov 1;132(21):e150750. doi: 10.1172/JCI150750 (PMC9621138; doi:10.1172/JCI150750)
Supplement: Supplemental data [file jci-132-150750-s008.pdf]

Supplemental Information

CD200 ectodomain shedding into the tumor microenvironment leads to NK cell dysfunction and apoptosis

Huw J. Morgan[+1], Elise Rees[+1], Simone Lanfredini[1], Kate A. Powell[1], Jasmine Gore[1], Alex Gibbs[1], Charlotte Lovatt[1], Gemma E. Davies[1], Carlotta Olivero[1], Boris Y. Shorning[1], Giusy Tornillo[1], Alex Tonks[2], Richard Darley[2], Eddie C.Y. Wang[3], Girish K. Patel[1]

[1] European Cancer Stem Cell Research Institute, School of Biosciences, Cardiff University, Cardiff, UK.

[2] Department of Haematology, Division of Cancer & Genetics, School of Medicine, Cardiff University, Cardiff, UK

[3] Division of Infection and Immunity, Cardiff University School of Medicine, Cardiff, UK

[+] Contributed equally to this work

Lead Contact: Girish K Patel; Email: patelgk@cardiff.ac.uk

Supplemental Figure 1

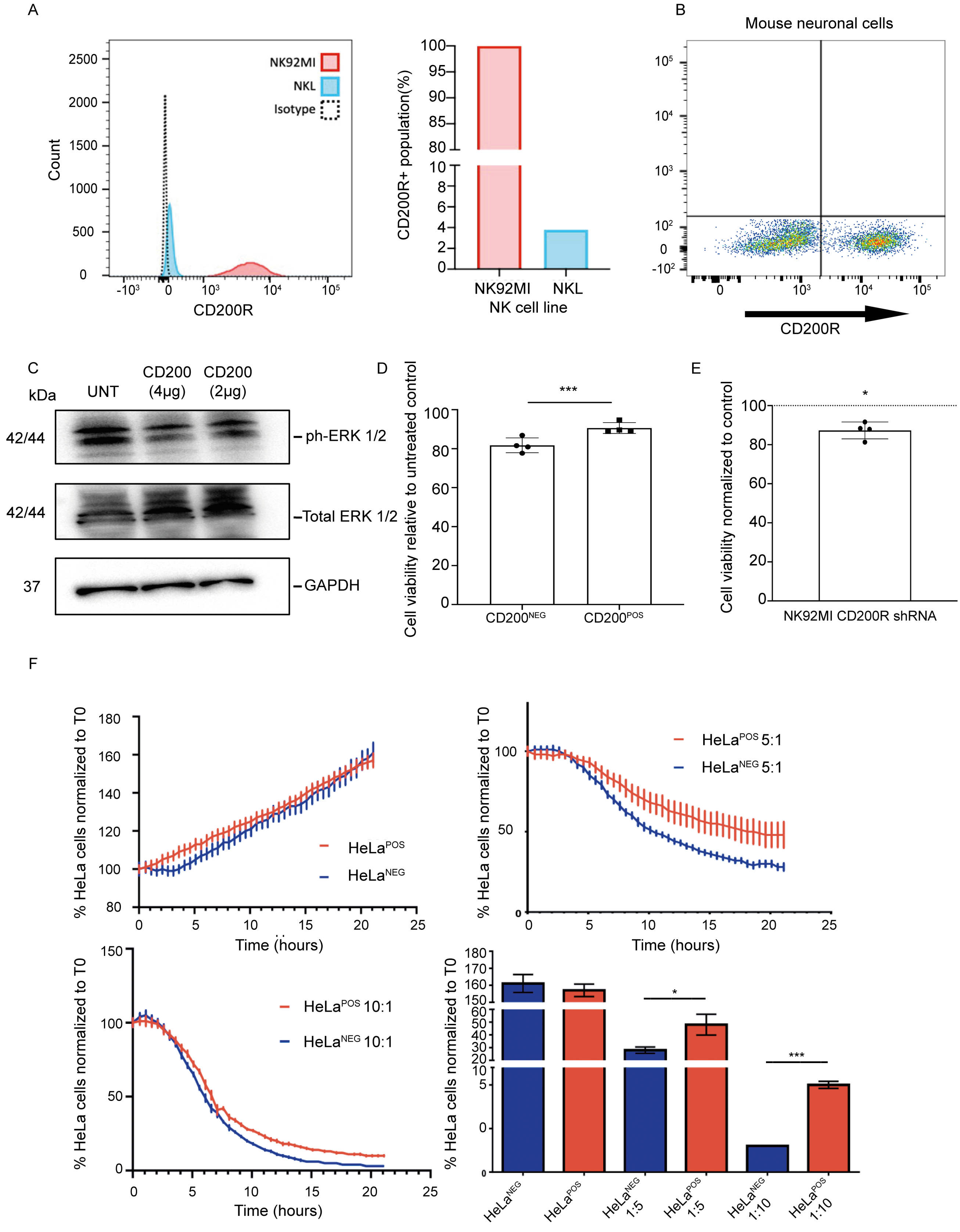

Supplemental Figure 2

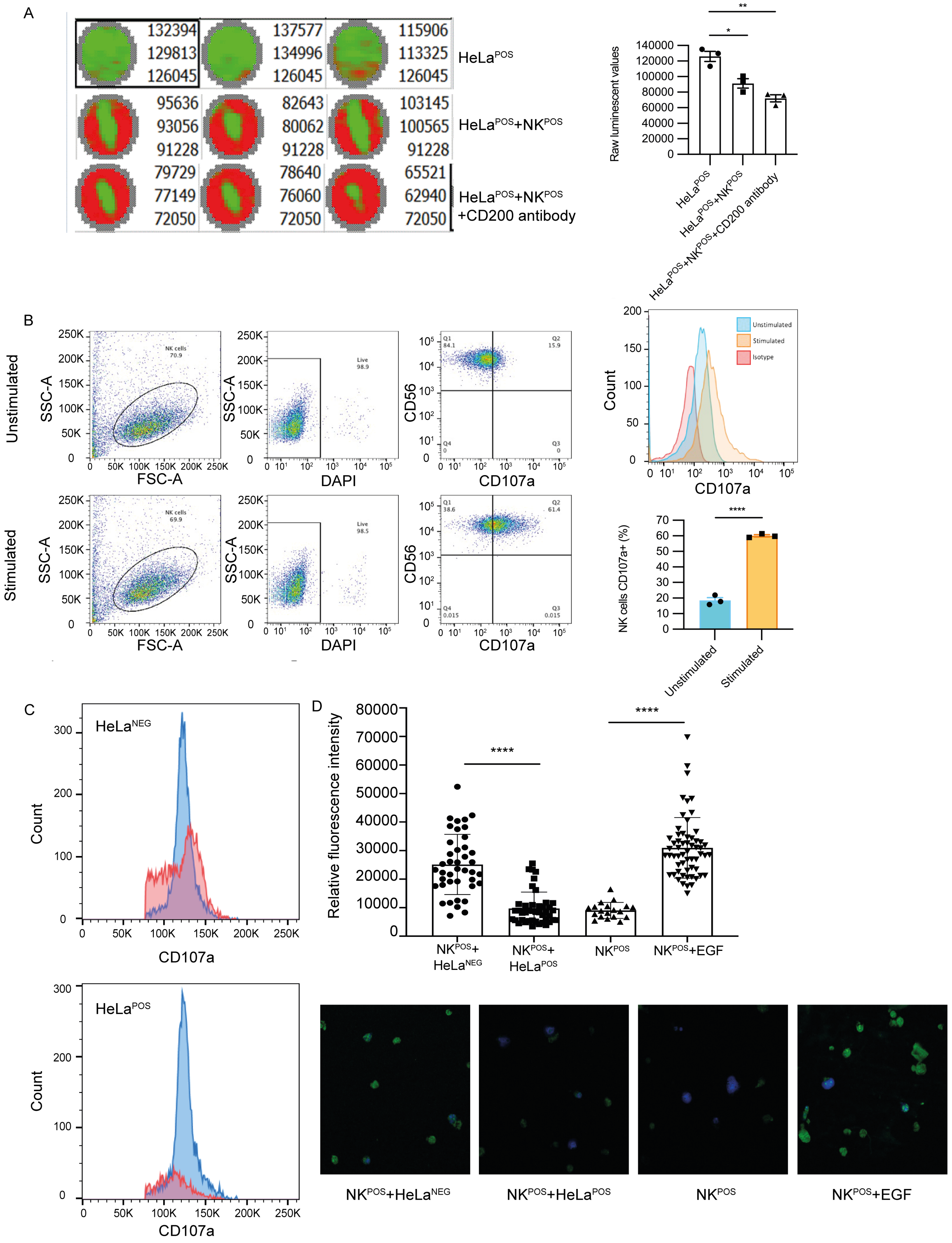

Supplemental Figure 3

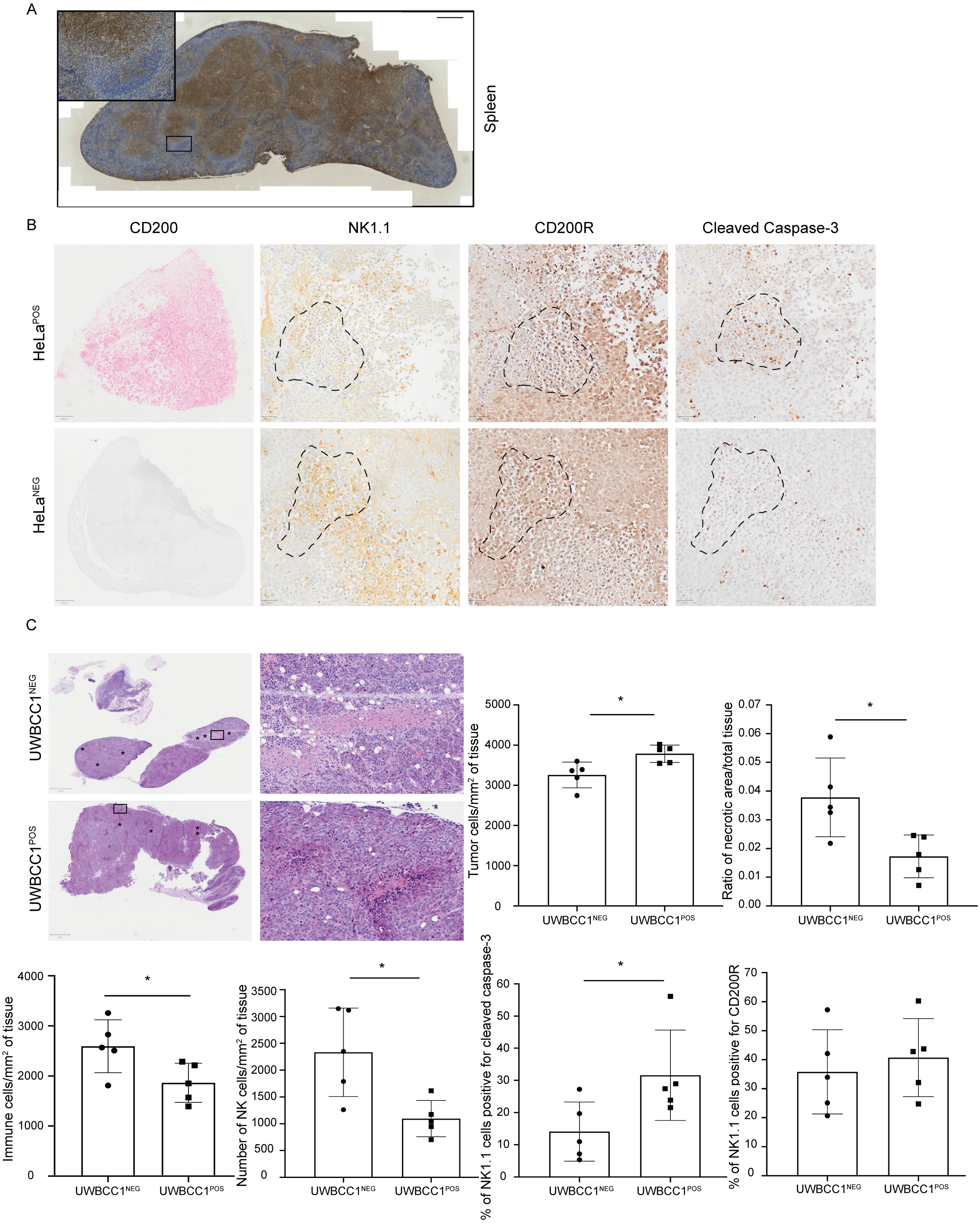

Supplemental Figure 4

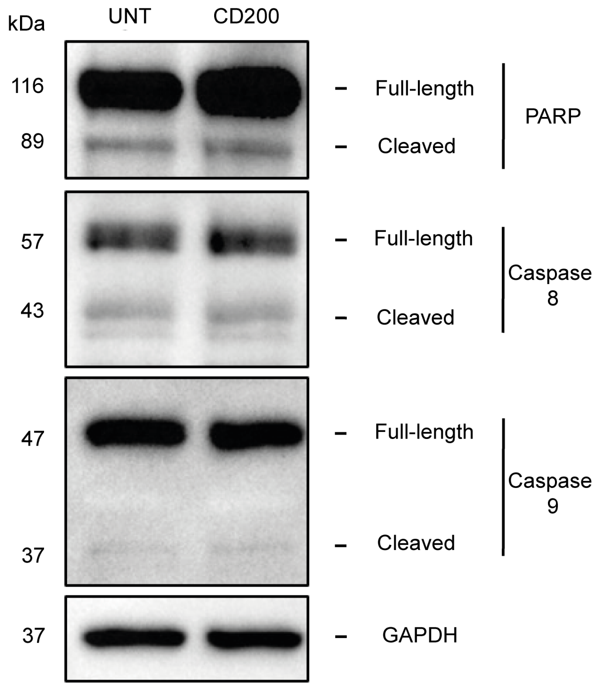

Supplemental Figure 5

A

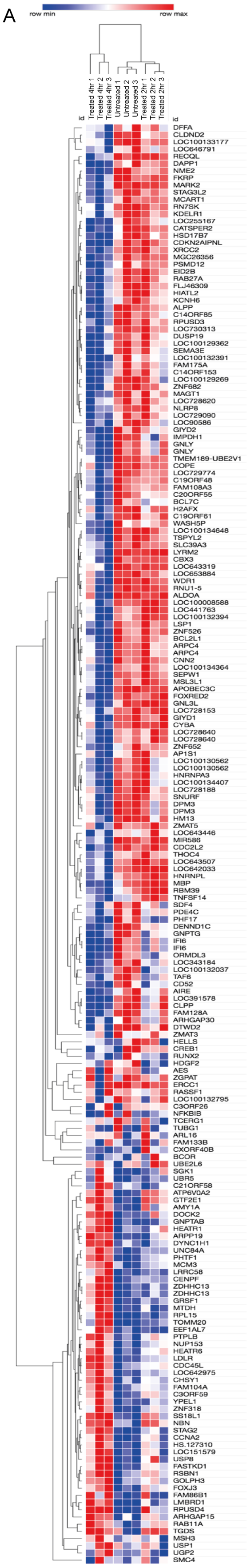

B

Gene Ontology Biological Processes	P values
**2 Hours**	
positive regulation of apoptotic process (GO:0043065)	9.69E-04
positive regulation of release of cytochrome c from mitochondria (GO:0090200)	0.002070705
mitochondrial fragmentation involved in apoptotic process (GO:0043653)	0.003320468
positive regulation of programmed cell death (GO:0043068)	0.004053175
regulation of release of cytochrome c from mitochondria (GO:0090199)	0.00711394
positive regulation of mitochondrial fission (GO:0090141)	0.008126029
release of cytochrome c from mitochondria (GO:0001836)	0.013314368
intrinsic apoptotic signaling pathway in response to DNA damage by p53 class mediator (GO:0042771)	0.016322711
mitochondrial fission (GO:0000266)	0.017925289
positive regulation of apoptotic signaling pathway (GO:2001235)	0.039182609
apoptotic mitochondrial changes (GO:0008637)	0.04406805
**4 Hours**	
negative regulation of execution phase of apoptosis (GO:1900118)	0.00249665
regulation of execution phase of apoptosis (GO:1900117)	0.006737694
release of cytochrome c from mitochondria (GO:0001836)	0.012804955
regulation of extrinsic apoptotic signaling pathway in absence of ligand (GO:2001239)	0.02775977
apoptotic mitochondrial changes (GO:0008637)	0.042469604

WikiPathway	P values
**2 Hours**	
HIF1A and PPARG regulation of glycolysis WP2456	0.07614882
Cori Cycle WP1946	0.007973
**4 Hours**	
Apoptosis WP254	0.0089008
DNA Damage Response WP707	0.02804896
DNA IR-Double Strand Breaks (DSBs) and cellular response via ATM WP3959	0.01607344
DNA IR-damage and cellular response via ATR WP4016	0.00751543
Fas Ligand (FasL) pathway and Stress induction of Heat Shock Proteins (HSP) regulation WP314	0.33481328

C

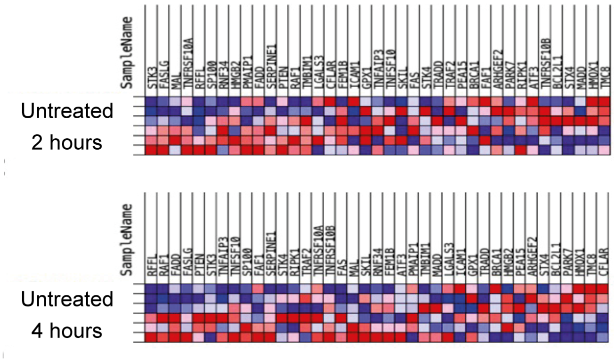

D

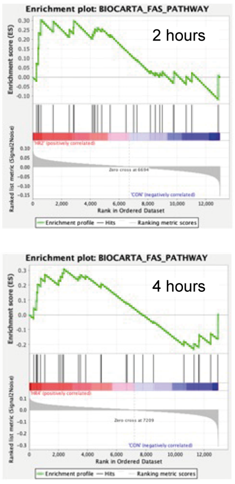

E

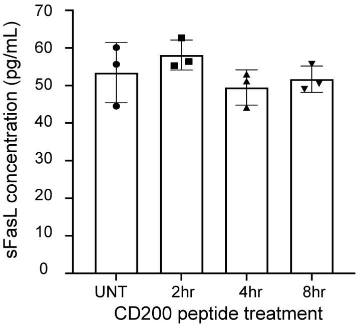

F

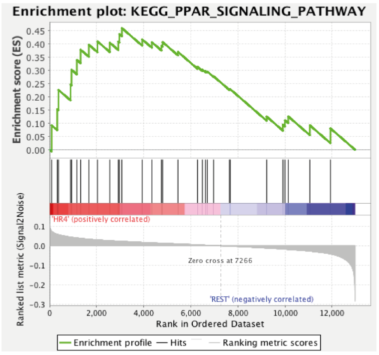

G

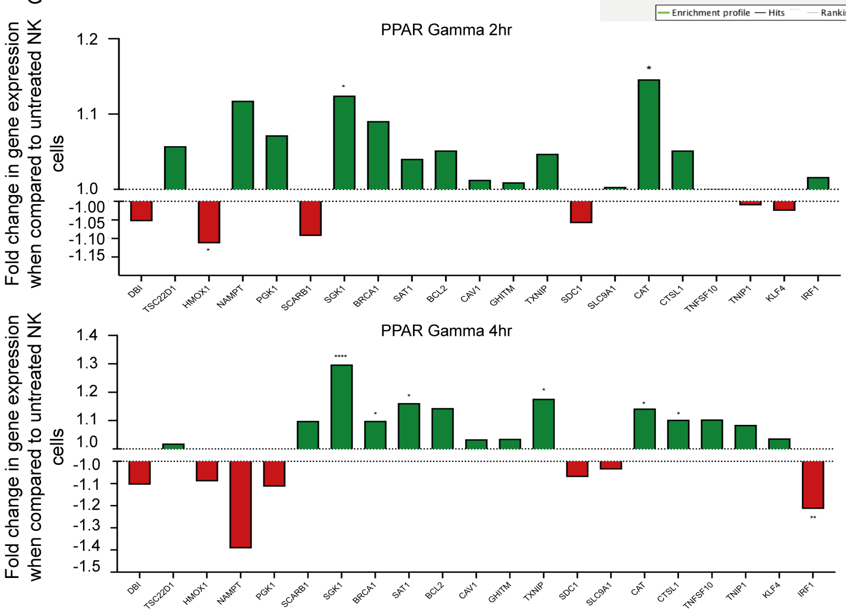

Supplemental Figure 6

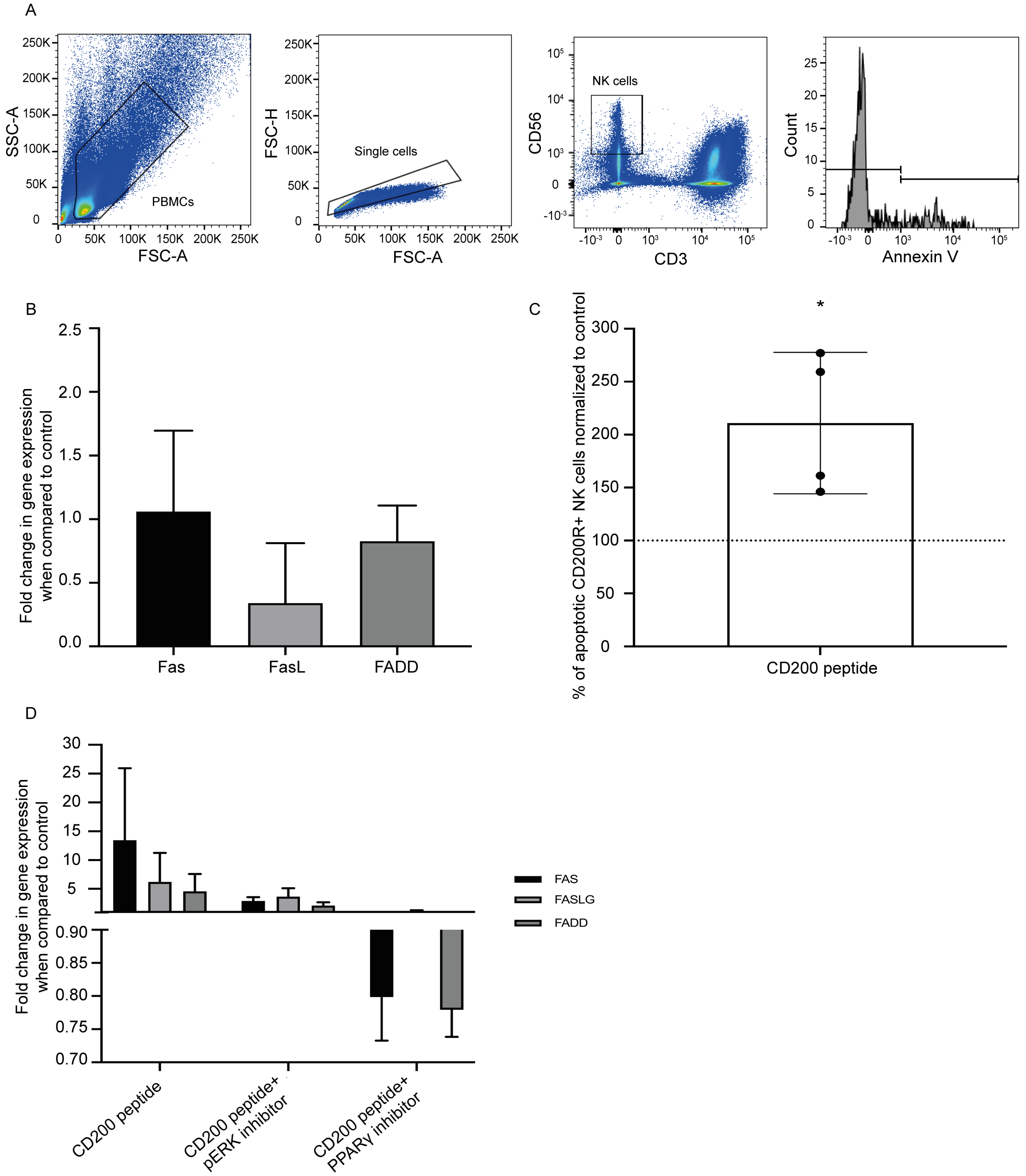

Supplemental Figure 7

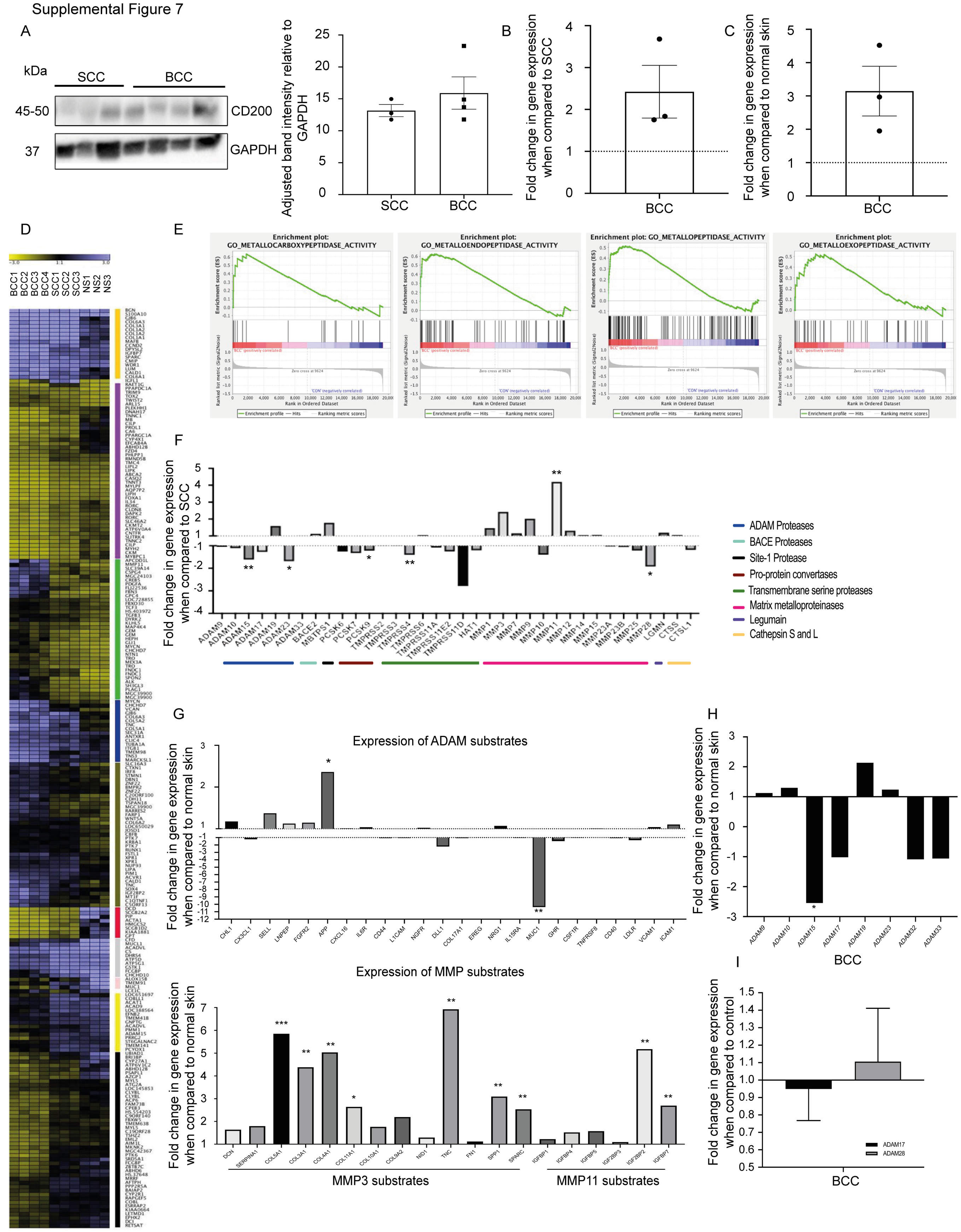

Supplemental Figure 8

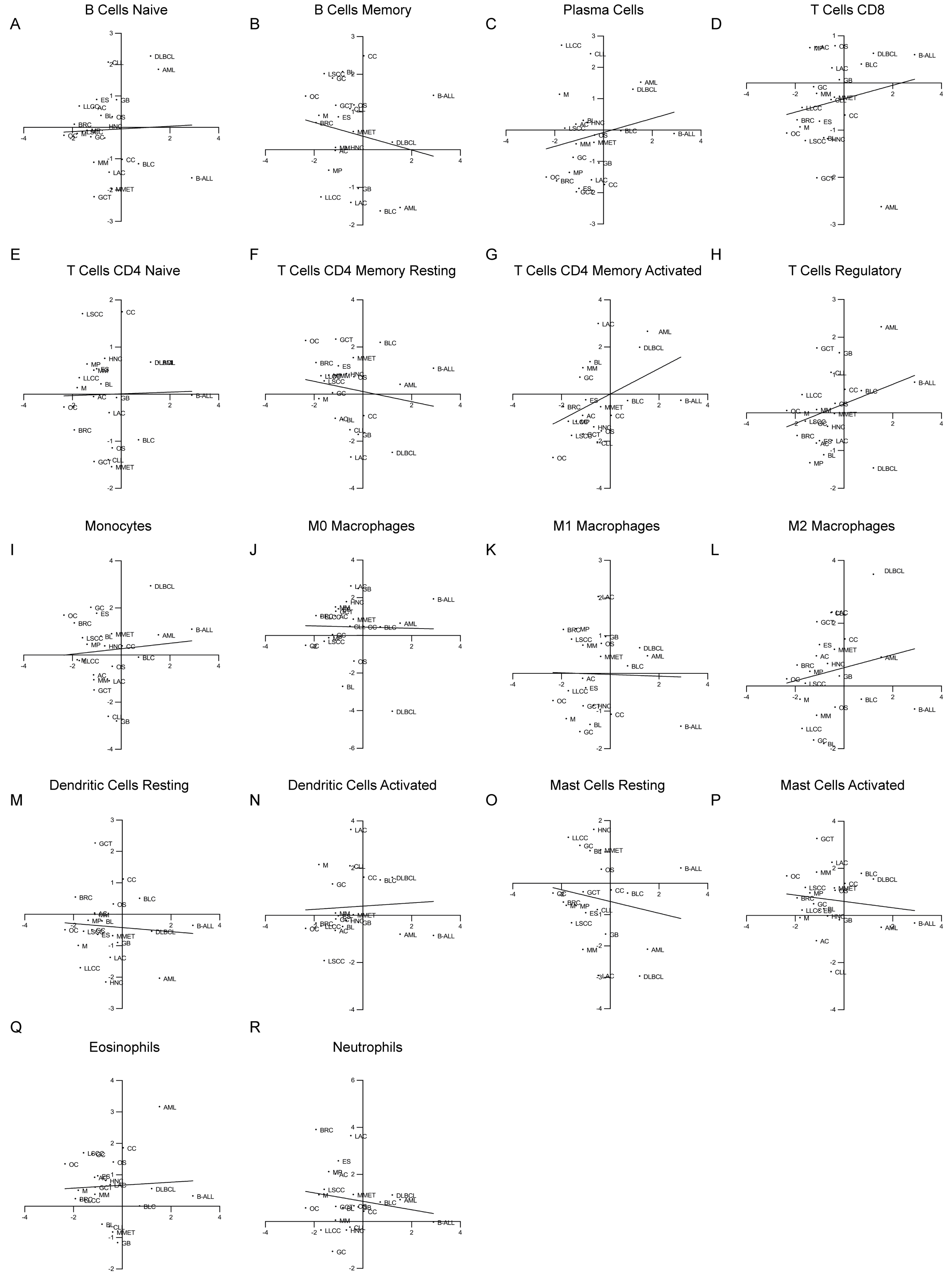

Supplemental Figure 9

A

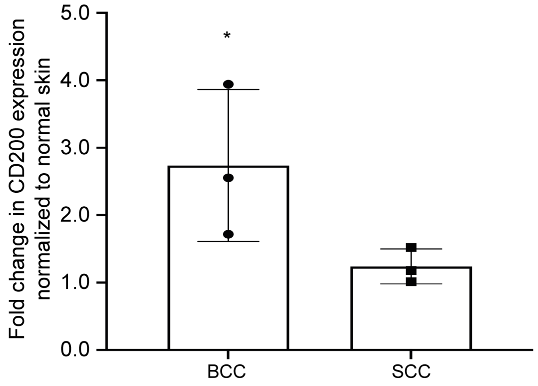

B

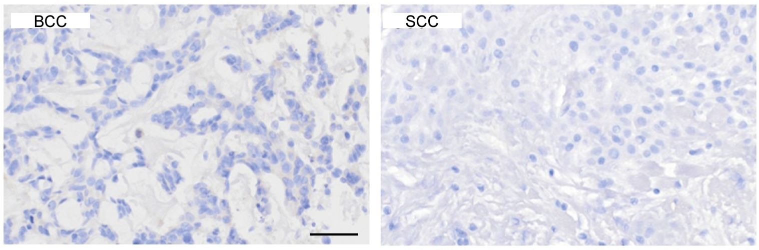

C

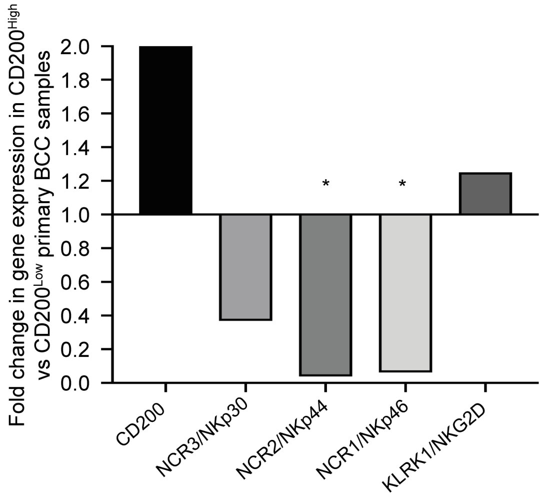

D

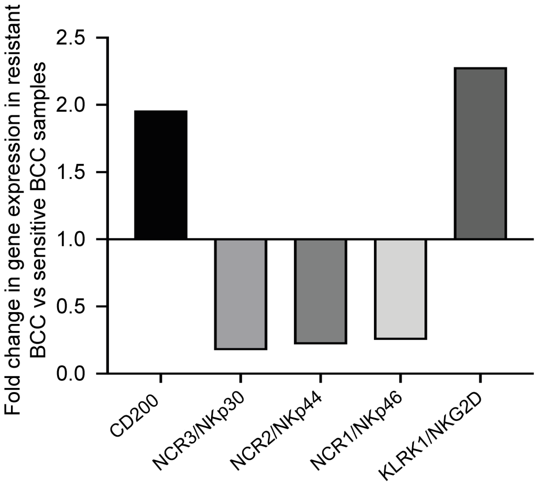

Supplementary Table 1

#	Gene	Fold Change	Gene	Fold Change
1	VCAN	3.14	DCD	-5.70
2	APCDD1L	2.97	PIP	-4.08
3	MYCN	2.93	SCGB2A2	-3.93
4	SLC39A14	2.82	ALOX15B	-3.39
5	FBN3	2.80	MUC1	-3.37
6	TNC	2.79	ACTA1	-3.19
7	COL1A1	2.76	TMEM91	-2.91
8	FLJ22536	2.61	KIA18881	-2.87
9	MMP11	2.61	SCGB1D2	-2.82
10	COL5A1	2.55	MUCL1	-2.80
11	COL1A2	2.55	CFD	-2.74
12	CHCHD7	2.54	GPT	-2.59
13	COL5A2	2.51	HMGCS2	-2.59
14	GJB6	2.46	UBIAD1	-2.54
15	PDGFA	2.45	FCGBP	-2.52
16	LUM	2.44	PSAPL1	-2.45
17	CSPG4	2.42	BRI3BP	-2.34
18	IGF2BP2	2.37	AZGP1	-2.19
19	SPON2	2.33	CHCHD10	-2.14
20	CREB5	2.19	PROL1	-2.08
21	CALD1	2.17	FCGBP	-2.01
22	COL6A3	2.15		
23	COL1A2	2.15		
24	COL3A1	2.13		

Supplementary Table 2

**Tumor Type**	**CD200 Z-Scores**
Adrenocortical_cancer	-1.523
Bladder_cancer	0.71
Brain_cancer_Astrocytoma	-1.131
Brain_cancer_Glioblastoma	-0.192
Brain_cancer_Glioma	-4.949
Brain_cancer_Medulloblastoma	-0.774
Brain_cancer_Meningioma	-1.803
Brain_cancer_Neuroblastoma	-7.223
Breast_cancer	-1.924
Colon_cancer	0.041
Gastric_cancer	-1.247
Germ_cell_tumors	-1.113
Head_and_neck_cancer	-0.674
Head_and_neck_cancer_Hypopharyngeal_cancer	0.646
Head_and_neck_cancer_Oesophageal_cancer	0.9
Head_and_neck_cancer_Oral_SCC	0.455
Hematopoietic_cancer_AML	1.529
Hematopoietic_cancer_B_ALL	2.899
Hematopoietic_cancer_Burkitt_lymphoma	-0.827
Hematopoietic_cancer_CLL	-0.533
Hematopoietic_cancer_DLBCL	1.207
Hematopoietic_cancer_FL	-0.24
Hematopoietic_cancer_Mantle_cell_lymphoma	-1.779
Hematopoietic_cancer_Multiple_myeloma	-1.122
Kidney_cancer	-1.247
Liver_cancer	1.022
Liver_cancer_Primary	0.028
Lung_cancer_ADENO	-0.492
Lung_cancer_LCC	-1.718
Lung_cancer_SCC	-1.587
Lung_cancer_SCLC	0.117
Melanoma	-1.399
Melanoma_Metastasis	-0.392
Mesothelioma	-1.601
Ovarian_cancer	-2.347
Pancreatic_cancer	0.544
Prostate_cancer	-0.585
Sarcoma_Ewing_sarcoma	-1.003
Sarcoma_Osteosarcoma	-0.366

Supplementary Table 3

	Z-Score																						
**Cancer Type**	**CD200**	**NK Cells Resting**	**NK Cells Activated**	**T Cells Gamma Delta**	**B Cells Naïve**	**B Cells Memory**	**Plasma Cells**	**T Cells CD8**	**T Cells CD4 Naïve**	**T Cells CD4 memory resting**	**T Cells CD4 memory activated**	**T Cells follicular helper**	**T Cells regulatory**	**Monocytes**	**M0 Macrophages**	**M1 Macrophages**	**M2 Macrophages**	**Dendritic Cells resting**	**Dendritic cells activated**	**Mast Cells resting**	**Mast cells activated**	**Eosinophils**	**Neutrophils**
**AML**	1.529	-1.49	1.98	-1.61	1.85	-1.53	1.53	-2.62	0.68	0.42	2.67	-2.38	2.28	0.86	0.65	0.47	0.92	-2.03	-0.80	-2.10	-0.50	3.17	0.93
**B-ALL**	2.899	-1.16	1.49	0.05	-1.61	1.44	-0.11	0.60	-0.02	1.10	-0.26	-2.79	0.81	1.10	1.94	-1.40	-0.73	-0.35	-0.85	0.49	-0.32	0.33	-0.02
**CLL**	-0.533	-2.39	-0.54	-1.83	2.08	1.09	2.44	-0.35	-1.39	-1.51	-2.06	0.69	1.07	-2.60	0.49		2.34		2.10	-0.84	-2.38	-0.65	-0.24
**Burkitt's Lymphoma**	-0.827	0.93	-0.75	-1.35	0.38	2.07	0.31	-1.16	0.22	-1.07	1.38	-2.21	-1.11	0.81	-2.71	-1.35	-1.83	-0.21	-0.47	1.04	0.29	-0.57	0.58
**DLBCL**	1.207	0.91	1.24	-1.70	2.28	0.20	1.30	0.63	0.68	-2.47	1.99	-3.33	-1.46	2.94	-4.03	0.68	3.56	-0.54	1.61	-2.96	1.55	0.56	1.13
**Multiple Myeloma**	-1.122	1.72	1.82	1.67	-1.11	0.06	-0.44	-0.22	0.51	0.81	1.12	0.01	0.09	-1.05	1.51	0.75	-0.93	0.00	0.10	-2.11	1.84	0.38	0.06
**Astrocytoma**	-1.131	1.05	0.08	1.28	0.64	-0.02	0.19	0.77	-0.05	-1.02	-0.90	1.75	-0.80	-0.84	1.03	-0.13	0.96	0.04	-0.64		-1.07	0.92	2.02
**Glioblastoma**	-0.192	0.70	-0.02	1.37	0.88	-1.03	-1.05	0.07	-0.07	-1.71		3.11	1.59	-2.80	2.49	0.98	0.32	-0.90	-0.29	-1.62	-0.18	-1.16	0.62
**Meningioma**	-1.803	1.62	-1.78	0.70	-0.19	0.91	1.14	-0.93	0.14	-0.19		-0.75	0.00	-0.20		-1.20	-0.42	-0.99	2.15	-0.70	-0.09	0.51	1.14
**Bladder Cancer**	0.71	1.79	-0.29	-2.07	-1.16	-1.62	-0.02	0.40	-0.97	2.20	-0.27	-0.89	0.59	-0.09	0.46	0.20	-0.42	0.51	1.50	-0.31	1.79	0.01	0.83
**Breast Cancer**	-1.924	1.34	-1.51	-1.28	0.10	0.72	-1.62	-0.78	-0.76	1.34	-0.52	-0.24	-0.60	1.37	1.05	1.17	0.67	0.55	-0.33	-0.60	0.75	0.24	3.91
**Colon Cancer**	0.041	-0.31	1.77	-1.80	-1.02	2.49	-1.74	-0.68	1.75	-0.91	-0.90	0.21	0.62	0.39	0.47	-1.08	1.50	1.12	1.62	-0.21	1.36	1.86	0.44
**Ewing Sarcoma**	-1.003	-0.23	-1.27	-0.61	0.89	0.87	-1.86	-0.81	0.54	1.20	-0.24	-1.28	-0.74	1.78	1.41	-0.38	1.32	-0.64	-0.02	-0.94	0.22	0.97	2.58
**Gastric Cancer**	-1.247	0.00	-1.04	0.99	-0.30	1.90	-0.87	-0.08		0.06	0.72	0.88	-0.27	2.03	0.04	-1.54	-1.72	-0.49	1.33	1.21	0.50	1.66	-1.27
**Germ Cell Tumours**	-1.113	1.30	-0.50	-1.40	-2.21	1.18	-1.97	-2.01	-1.43	2.34	-1.69	-2.70	1.72	-1.48	1.28	-0.86	2.05	2.27	-0.15	-0.27	3.27	0.61	0.64
**Head and Neck Cancer**	-0.674	0.18	-1.46	-1.81	-0.34	0.07	0.24	-1.18	0.76	0.87	-1.39	0.51	-0.36	0.39	1.79	-0.85	0.72	-2.15	-0.21	1.72	-0.02	0.82	-0.36
**Lung Adenocarcinoma**	-0.492	1.23	0.25	-0.54	-1.43	-1.40	-1.59	0.32	-0.39	-2.67	3.00	-0.99	-0.73	-1.09	2.62	2.04	2.36	-1.37	3.64	-2.94	2.26	0.69	3.65
**Lung SCC**	-1.587	1.07	-0.26	-1.23	-0.13	2.02	0.06	-1.22	1.71	0.56	-1.75	-0.51	-0.22	0.74	-0.31	0.91	0.09	-0.54	-1.92	-1.26	1.19	1.70	1.37
**Lung Large Cell Carcinoma**	-1.718	-0.24	-0.28	-2.04	0.68	-1.25	2.71	-0.52	0.35	0.78	-1.15	-0.35	0.48	-0.23	1.01	-0.46	-1.35	-1.70	-0.45	1.47	0.23	0.20	-0.36
**Melanoma Primary**	-1.399	1.01	0.24	-1.29	-0.09	-0.54	-1.35	0.75	0.64	0.81	-1.15	1.86	-1.32	0.46	-0.12	1.19	0.47	-0.19		-0.71	0.96		2.12
**Melanoma Metastasis**	-0.392	0.59	-1.14	-0.79	-1.96	0.48	-0.39	-0.30	-1.54	1.55	-0.53	-1.54	-0.02	0.91	0.86	0.45	1.17	-0.68	0.03	1.07	1.17	-0.82	1.15
**Osteosarcoma**	-0.366	0.71	0.56	-0.82	0.34	1.19	-0.16	0.79	-1.14	0.74	-1.56	1.36	0.25	-0.48	-1.36	0.80	-0.67	0.33		0.45	1.07	1.41	0.67
**Ovarian Cancer**	-2.347	-0.42	0.84	1.02	-0.25	1.42	-1.50	-1.06	-0.27	2.28	-2.69	-0.73	0.06	1.70	-0.53	-0.72	0.23	-0.49	-0.55	-0.31	1.68	1.35	0.58

Supplementary Table 4 - Resources Table

**REAGENT or RESOURCE**	**SOURCE**	**IDENTIFIER**
**Antibodies (Clone)**		
anti-human CD207 (9H)	Beckman Coulter	Cat# IM3449; RRID: AB_131719
anti-human CD123 (9F5)	BD Biosciences	Cat# 555642; RRID: AB395999
anti-human CD8 (C8/144B)	Dako	Cat# M7103; RRID: AB_2075537
anti-human CD4 (SK3)	BD Biosciences	Cat# 346320; RRID: AB_400250
anti-human CD14 (MφP9)	BD Biosciences	Cat# 347490; RRID: AB_400310
anti-human CD56 (NCAM16.2)	BD Biosciences	Cat# 559043; RRID: AB_397180
anti-human HLA-DR (L243)	BD Biosciences	Cat#347360; RRID: AB_400289
anti-human Cytokeratin 14 (LL002)	Abcam	Cat#ab7800; RRID: AB_306091
anti-human CD56-FITC (REA196)	Miltenyi Biotec	Cat#130-100-683; RRID: AB_2658729
anti-human CD3-APC (HIT3a)	BioLegend	Cat#300312; RRID: AB_314048
anti-human CD45-PE (HI30)	BD Biosciences	Cat#560975; RRID: AB_2033960
7AAD	BD Biosciences	Cat#559925
anti-human CD200 (OX-104)	BioLegend	Cat#329207; RRID: AB_2074064
anti-human CD107a (H4A3)	BD Biosciences	Cat#562622; RRID: AB_2737684

anti-human CD200R (OX-108)	BioLegend	Cat#329306; RRID: AB_2074200
anti-mouse NK1.1 (PK136)	ThermoFisher Scientific	Cat#MA1-70100; RRID: AB_2296673
DAPI	Sigma-Aldrich	Cat#10236276001
AF647 IgG1 isotype	BioLegend	Cat#MCA1209A647; RRID: AB_322324
PE Mouse IgG1, k Isotype Ctrl Antibody (MOPC-21)	BioLegend	Cat#400112; RRID: AB_2847829
APC Mouse IgG2a, k Isotype Ctrl Antibody (MOPC-173)	BioLegend	Cat#400220; RRID: AB_326468
FITC Human IgG1 Isotype Ctrl Recombinant Antibody	BioLegend	Cat#403508; RRID: AB_2847831
Purified Mouse IgG1, k Isotype Control (MOPC-21)	BD Biosciences	Cat#555746; RRID: AB_396088
CD200	R&D Systems	Cat#AF2724; RRID: AB_416669
Phospho-p44/42 MAPK (Erk1/2) (Thr202/Tyr204)	Cell Signalling	Cat#9101; RRID: AB_331646
PARP (46D11)	Cell Signalling	Cat#9532; RRID: AB_659884
Caspase 8 (1C12)	Cell Signalling	Cat#9746; RRID: AB_2275120
Caspase 9	Cell Signalling	Cat#9502; RRID: AB_2068621
Fas (4C3)	Cell Signalling	Cat#8023; RRID: AB_10860778
FasL	Cell Signalling	Cat#4273; RRID: AB_2100652
FADD	Cell Signalling	Cat#2782; RRID: AB_2100484

GAPDH	Merck Millipore	Cat#MAB374; RRID: AB_2107445
Cleaved caspase-3	Cell Signalling	Cat#9661
CD200R	Abcam	Cat#ab198010
CD200	R&D Systems	Cat#AF2724
NK1.1	ThermoFisher Scientific	Cat#MA1-70100
Horse Anti-Goat IgG Antibody (H+L), Biotinylated, R.T.U.	Vector Laboratories	BP-9500-50
Goat Anti-Rabbit IgG Antibody (H+L), Biotinylated, R.T.U.	Vector Laboratories	BP-9100-50
Goat Anti-Mouse IgG Antibody (H+L), Biotinylated, R.T.U.	Vector Laboratories	BP-9200-50
Donkey anti-Goat IgG (H+L) Cross-Adsorbed Secondary Antibody, Alexa Fluor 488	Invitrogen	Cat#A-11055; RRID: AB_2534102
Donkey anti-Rabbit IgG (H+L) Highly Cross-Adsorbed Secondary Antibody, Alexa Fluor 568	Invitrogen	Cat#A-10042; RRID: AB_2534017
Donkey anti-Mouse IgG (H+L) Highly Cross-Adsorbed Secondary Antibody, Alexa Fluor 647	Invitrogen	Cat#A-31571; RRID: AB_162542

Goat Anti-Rabbit IgG H&L (HRP)	Abcam	Cat#ab97051; RRID: AB_10679369
Goat Anti-Mouse IgG1 (HRP)	Abcam	Cat#ab98693; RRID: AB_10674928
Goat Anti-Mouse IgG2a heavy chain (HRP)	Abcam	Cat#ab97245; RRID: AB_10680049
Rabbit Anti-Goat IgG H&L (HRP)	Abcam	Cat#ab97100; RRID: AB_10687752
**Chemicals, Peptides and Recombinant Proteins**		
Recombinant Human CD200 Peptide	R&D Systems	Cat#2724-CD-050
Recombinant Mouse CD200 Peptide	R&D Systems	Cat#3355-CD-050
Purified Mouse Anti-Human CD200	BD Biosciences	Cat#552023
Recombinant Human MMP3 protein	R&D Systems	Cat#513-MP-010
Recombinant Human MMP11 (Catalytic domain)	Enzo Life Sciences	Cat#BML-SE282-0010
Recombinant Human TIMP-3 Protein	R&D Systems	Cat#973-TM
Bovine Pituitary Extract	ThermoFisher Scientific	Cat#13028014
Recombinant Human Fibroblast Growth Factor	PeproTech Ltd	Cat#AF-100-18B
Recombinant Human Epidermal Growth Factor	PeproTech Ltd	Cat#AF-100-15

Phorbol 12-myristate 13-acetate (PMA)	Abcam	Cat#ab120297
Ionomycin Ca2+ Salt, Ca^{2+} ionophore	Abcam	Cat#ab120116
Z-VAD-FMK (Pan-caspase inhibitor)	Selleckchem	Cat#S8102
Z-IETD-FMK (Caspase 8 inhibitor)	Selleckchem	Cat#S7314
Z-LEHD-FMK (Caspase 9 inhibitor)	Selleckchem	Cat#S7313
ZB4 clone anti-Fas mAb	Merck Millipore	Cat#05-338
GW9662 (PPARγ antagonist)	Selleckchem	Cat#S2915
Dispase	Worthington	Cat#LS02104
Collagenase III	Worthington	Cat#LS004182
Hyaluronidase	Worthington	Cat#LS005475
DNase I	Stem Cell Technologies	Cat#07900
Donkey Serum	Sigma-Aldrich	Cat#D9663
VECTASHIELD® Antifade Mounting Medium	Vector Laboratories	Cat#H-1000-10
Protease Inhibitor Cocktail (100x)	Cell Signalling	Cat#5871S
QuantiTect Reverse Transcription Kit	Qiagen	Cat#205313
TaqMan® Universal Master Mix II	ThermoFisher Scientific	Cat#4440040
Bovine Serum Albumin Powder	Fisher Bioreagents	Cat#BP9702-100
IncuCyte® Caspase-3/7 Reagent for Apoptosis	Essen Bioscience	Cat#4704
TWEEN®20	Sigma-Aldrich	Cat#P9416
IGEPAL®CA-630	Sigma-Aldrich	Cat#I8896

Immobilon Forte Western HRP substrate	Merck Millipore	Cat#WBLUF0100
Illumina™ TotalPrep™ RNA Amplification Kit	ThermoFisher Scientific	Cat#AMIL1791
Illumina HumanHT-12 v4 Expression BeadChip	Illumina	Cat#BD-901-1001
RPMI 1640 Medium	ThermoFisher Scientific	Cat#11875093
DMEM Medium	ThermoFisher Scientific	Cat#11960085
Keratinocyte Serum Free Media	ThermoFisher Scientific	Cat#17005059
Fetal Bovine Serum	ThermoFisher Scientific	Cat#10270106
Horse Serum, Heat Inactivated	ThermoFisher Scientific	Cat#26050088
Ficoll® Paque Plus	GE Healthcare	Cat#GE17-1440-02
Trans-Blot Turbo RTA Mini 0.2 µm PVDF Transfer Kit, for 40 blots	Bio-Rad	Cat#1704272
TGX™ FastCast™ Acrylamide Kit, 7.5%	Bio-Rad	Cat#1610171
96 well plates, flat bottomed, white walled	Greiner Bio-One	Cat#655098
Pierce™ Protein Concentrator PES, 3K MWCO	ThermoFisher Scientific	Cat#88526
Pierce™ BCA Protein Assay Kit	ThermoFisher Scientific	Cat#23225
RetroNectin® Recombinant Human Fibronectin Fragment	TaKaRa Bio	Cat#T100A
**Complementary DNA**		
CD200	Gift of Alex Tonks, Department of	IMAGE consortium; Clone ID 5299899

	Haematology, Cardiff University, UK	
**Retroviral Expression Vector**		
PINCO	Gift of Alex Tonks, Department of Haematology, Cardiff University, UK	
**Critical Commercial Assays**		
RNeasy Plus Mini Kit	Qiagen	Cat#74134
CellTiter-Glo® Luminescent Cell Viability Assay	Promega	Cat#G7572
CD200 Matched ELISA Antibody Pair Set,Human	Sino Biological	Cat#SEK10886
Human CCL4/MIP-1 beta DuoSet ELISA kit	R&D Systems	Cat#DY271
Human Fas Ligand/TNFSF6 ELISA Kit	R&D Systems	Cat#DY126
Human IFN-gamma ELISpot kit	R&D Systems	Cat#EL285
VECTASTAIN® Elite ABC-HRP Kit, Peroxidase	VECTOR Laboratories	Cat#PK-6100
**Biological Samples**		
Fresh human tumour and PBMC samples	Hywel Dda and Cardiff and Vale University Health Boards	
Paraffin embedded human tumour samples	Hywel Dda and Cardiff and Vale University Health Boards	

Primary Mouse Neuronal Cells	Kindly provided by Niels Haan, Neuroscience and Mental Health Research Institute, Cardiff University, UK	
**Experimental Models: Cell Lines**		
HeLa	ATCC®	CCL-2™
NK92-MI	ATCC®	CRL-2408™
NKL	Gifted to Edward Wang from Michael Robertson	
NIH/3T3	ATCC®	CRL-1658™
Primary CD19+ B Cells	ATCC®	PCS-800-018™
UWBCC-1	Gifted from Vladimir Spiegelman	
**Experimental Models: Organisms/Strains**		
NU(NCr)-*Foxn1^{nu}* mice	Charles River, UK	
**Oligonucleotides (TaqMan)**		
CD200 (FAM-MGB)	ThermoFisher Scientific	Cat#Hs01033303_m1
FAS (FAM-MGB)	ThermoFisher Scientific	Cat#Hs00236330_m1
FASLG (FAM-MGB)	ThermoFisher Scientific	Cat#Hs00181225_m1
FADD (FAM-MGB)	ThermoFisher Scientific	Cat#Hs00538709_m1
ADAM17 (FAM-MGB)	ThermoFisher Scientific	Cat#Hs01041915_m1
ADAM28 (FAM-MGB)	ThermoFisher Scientific	Cat#Hs00248020_m1
MMP3 (FAM-MGB)	ThermoFisher Scientific	Cat#Hs00968305_m1
MMP11 (FAM-MGB)	ThermoFisher Scientific	Cat#Hs00968295_m1
ACTB (FAM-MGB)	ThermoFisher Scientific	Cat#Hs00357333_g1
GAPDH (FAM-MGB)	ThermoFisher Scientific	Cat#Hs02758991_g1
**Software and Algorithms**		
CIBERSORT	Newman *et al.,* 2015	https://cibersort.stanford.edu
FlowJo v10.6.0		https://www.flowjo.com

GraphPad Prism v8		https://www.graphpad.com
Image J		https://imagej.nih.gov/ij/
Qupath	Bankhead *et al*., 2017	https://qupath.github.io
ImageLab		https://www.bio-rad.com
PRECOG	Gentles *et al.,* 2015	https://precog.stanford.edu
GSEA	Subramanian *et al*., 2005; Mootha *et al*., 2003	https://www.gsea-msigdb.org/gsea/index.jsp
R v3.4		https://cran.r-project.org
**Other**		
BD LSRFortessa	BD Biosciences	
Axio Scan.Z1 Slide Scanner	Zeiss	
QuantStudio™ 7 Flex Real-Time PCR System	ThermoFisher Scientific	
iScan Microarray Scanner	Illumina	
GelCount™ Colony Counter	Oxford Optronix	
CLARIOstar plate reader	BMG LABTECH	

***Primary antibodies used in flow cytometry**

Supplementary Figure 1. CD200R activation blocks MAPK signaling and NK cell killing activity

Flow cytometric analysis of NK cell lines (A) and mouse neuronal cells for CD200R expression (B). Immunoblot of lysates from mouse neuronal cells incubated for 10 minutes with CD200 peptide and probed for phosphorylated ERK1/2, total ERK and GAPDH (C). HaCaT cell transduced with CD200 and control vector, CD200^{POS} and CD200^{NEG} respectively, viability after incubation with The NK^{POS} cell line (D). CD200^{POS} HaCaT cells were co-cultured with NK^{POS} cell line treated with CD200R ShRNA or vector (E). Time-lapse quantification of viable GFP-HeLa (target cell) cells in co-culture with NK^{POS} (effector) cell at an E:T ratio of 0:1, 5:1 and 10:1 (n=3) over 20 hours (F). Error bars represent mean ± SEM. Significance values, *<0.05, ***<0.001.

Supplementary Figure 2. Immune cell activation is impaired by CD200.

Clariostar™ quantification of CD200^{POS} HaCaT cells alone, co-cultured with NKPOS cell line and treated with CD200 blocking antibody (A). The NK^{POS} cell line was stimulated with PMA and Ionomycin for 4 hr (B). Unstimulated NK^{POS} cells were used as a baseline. The expression of CD107a was analyzed in live, CD56+ NK^{POS} cells by flow cytometry (n=3). Average CD107a positivity was calculated. Students t test was used to determine difference between CD107 population positivity, **** represents p<0.0001. Error bars represent mean ± SEM. Flow cytometric histogram analysis of the lymphocyte population forward scatter comparing day 0 (blue histogram) and 5 (red histogram) blood samples from HeLa^{NEG} and HeLa^{POS} grafted mice (C). Immunofluorescence labelling for phospho-ERK1/2 and DAPI to determine MAPK pathway activation in NK^{POS} cells: untreated, treated with epidermal

growth factor (EGF) for 4 hours, and after 4 hour co-cultured with $HeLa^{NEG}$ and $HeLa^{POS}$ (D). Error bars represent mean ± SEM. Significance values, *<0.05, ****<0.0001.

Supplementary Figure 3. CD200 blocks NK cell killing

Paraffin embedded sections labelled with anti-NK1.1 antibody by immunohistochemistry to determine NK cell infiltrate, used spleen sections as positive control (A). Paraffin embedded sections were also labelled with anti-cleaved caspase 3 and -CD200R antibodies by immunohistochemistry to determine the fraction of NK cells undergoing apoptosis and the proportion of remaining NK cells that expressed the CD200 receptor, respectively (B). Week 5 tumors from nude mice were grafted with $3x10^6$ $UWBCC1^{POS}$ or $UWBCC1^{NEG}$ (n=5 each), histological analysis of tumor cellularity, necrosis and inflammatory cell infiltration, and data from paraffin embedded sections labelled with anti-NK1.1, -cleaved caspase 3 and -CD200R antibodies by immunohistochemistry (C). *=necrotic tissue area. Error bars represent mean ± SEM. Significance values, *<0.05.

Supplementary Figure 4. NK^{NEG} cells incubated with CD200 peptide

Immunoblot of CD200 peptide (4 µg/$1x10^6$ cells for 8-hours) incubated NK^{NEG} cells lysates for PARP, caspase 8, caspase 9 and GAPDH.

Supplementary Figure 5. Over-expression of the Fas death receptor extrinsic apoptotic pathway in NK cells treated with CD200 peptide.

Hierarchical clustering of differentially expressed genes from NK^{POS} cells incubated with CD200 peptide for 2- and 4-hours compared to untreated cells (A). Gene set enriched

analysis using Gene Ontology and WikiPathway terms (B). The Gene Ontology term "regulation of extrinsic apoptosis signaling pathway via death domain receptors" (GO:1902041) was identified as enriched, with 39 of the 58 genes within the gene set found to be concordantly regulated after 2 hours (left hand side) and 4 hours (right hand side) (C). GSEA of the Biocarta "Fas Pathway" from differentially expressed genes comparing untreated and 2-hour (left panel) or 4-hour (right panel) CD200 peptide (4 μg/1x10^6 cells) treated NK^{POS} cells (D). ELISA determination of soluble FasL in culture supernatant of CD200 peptide treated NK^{POS} cells (E). GSEA of the Kegg "PPAR signaling Pathway" from differentially expressed genes comparing untreated and 2-hour (left panel) or 4-hour (right panel) CD200 peptide (4 μg/1x10^6 cells) treated NK^{POS} cells (F). Relative expression of PPARϒ regulated genes comparing untreated and 2-hour (above panel) or 4-hour (below panel) CD200 peptide (4 μg/1x10^6 cells) treated NK^{POS} cells (G). Green bars are concordant genes, discordant regulated genes are red bars. Error bars are represented as mean ± SEM.

Supplementary Figure 6. CD200 induced NK cell apoptosis.

PBMCs were extracted from whole blood (n=4) using Ficoll-Paque separation then plated for overnight to remove adherent cells. The non-adherent population was treated with CD200 peptide for 4-hours before FACs analysis (A). RNA extracted from CD200 peptide treated CD45+CD3- CD56+ CD200R+ and - flow sorted cells was analysed for FAS, FASL and FADD gene expression (B). Annexin V labelling was used to determine the frequency of apoptotic cells within the CD45+ CD3- CD56+ CD200R+ and CD45+ CD3- CD56+ CD200R- fractions (C). Analysis for FAS, FASL and FADD gene expression relative to untreated cells after treatment with CD200 peptide, as well as pharmacological inhibition of ERK and PPAR-γ in NK^{POS} cells (D). Significance values, *<0.05. Error bars represent mean ± SEM.

Supplementary Figure 7. CD200 ectodomain shedding

Primary human BCC (n=4) and SCC (n=3) tissue lysates were probed for CD200 and GAPDH and quantitated using ImageJ (A). qPCR relative expression of mRNA extracted from primary human BCC (n=3) and SCC (n=3) tissues, with GAPDH normalization (B). qPCR relative expression of mRNA extracted from primary human BCC (n=3) and matched normal skin (n=3) for CD200, with GAPDH normalization (C). Transcriptomic analysis of BCC (n=4), SCC (n=3) and normal skin (n=3) unsupervised hierarchical clustering revealed 235 differential expressed genes (D), with gene set enrichment for metallocarboxypeptidase activity and metalloendopeptidase activity (E). The relative gene expression for BCC versus SCC datasets for known sheddases were analyzed (F). The expression of the sheddase substrates, ADAMs, MMP3 and MMP11 were also analyzed for BCC versus normal skin (G). Similarly, we analyzed the relative expression of ADAMs (H) and validated the findings by qPCR of primary human BCC (n=4) for ADAM 17 and 28, with GAPDH normalization (I). Error bars represent mean ± SEM.

Supplementary Figure 8. Linear regression analysis of other immune cell phenotypes with CD200 prognostic z-scores.

The PRECOG and iPRECOG databases were used to generate data matrices for the associations, in the form of z-scores, between CD200 expression and overall cancer survival outcomes, and immune cell type and overall cancer survival outcomes, respectively. Briefly, PRECOG and iPRECOG databases were downloaded and filtered for CD200 expression and NK cell status and rows with NA values were discarded. The resulting data matrix was then imported into GraphPad and a Normal QQ plot and residual plot was generated to test for normality and variance in the data, respectively. Normality of the data was confirmed, and

assumptions of equal variance was met, and simple linear regressions were plotted for each graph (A-R).

Supplementary Figure 9. Basal cell carcinoma NK cell immune evasion.
Expression of CD200 within primary BCC (n=3) and SCC (n=3) tissue was established before grafting into mice (A). Paraffin embedded sections from primary BCC (n=3) and SCC (n=3) xenografts were labelled with anti-CD200 via IHC (B). Differentially expressed genes from a publicly available BCC dataset (GSE58377) were extracted to determine expression of NK cell receptor genes for $CD200^{High}$ vs $CD200^{Low}$ BCC (C) and resistant vs sensitive BCC samples (D). Significance values, *<0.05. Error bars represent mean ± SEM.

Supplementary Table 1. Top differentially expressed genes when comparing BCC to normal skin; significance p<0.01

Supplementary Table 2. Meta z-scores generated from 39 cancers to indicate prognosis from CD200 expression (+ and – values indicate poor and good prognosis, respectively)

Supplementary Table 3. Meta z-scores generated from 23 cancers to indicate prognosis between CD200 expression and immune cells (iPRECOG)

Supplementary Table 4. Resources Table

Supplemental Methods

Tissue dissociation and culture

Tissue for cell culture was processed into single cells as described in our previously published protocols (1,2).

Peripheral Blood Mononuclear Cell (PBMC) extraction for NK cell analysis

PBMC cells were extracted using Ficoll-Paque PLUS (3mL) solution as per manufacturers guidelines.

NK cell degranulation assay

NK92MI cells were co-cultured with HeLa cells as described above at an effector:target ratio of 5:1. As a positive control for degranulation, NK cells were treated with a combination of PMA (100ng/mL) and ionomycin (2μg/mL) (3). Untreated NK cells were used for baseline expression. The CD107a antibody was added at the beginning of the co-culture. Isotype control for CD107a was also added to the co-culture. After 30 mins of co-culture, 1x monensin was added to the well to prevent the reinternalization of the protein. The suspension NK92MI cells were removed from the co-culture and pelleted by centrifugation at 120xg for 5 mins. Cells were washed with FACS buffer (0.05% sodium azide and 0.5% BSA in PBS) before staining with CD56 antibody. Cells were washed and processed as described above.

NK92MI cell shRNA

200,000 NK-92MI cells (10^5 cells/ml) were transduced with lentiviral particles carrying shRNA against CD200 receptor (sc-42956-SH) or control shRNA (sc-42956-SH) in the presence of 8 μg/ml. Cells were then grown for 40 hours in fresh NK-92MI medium before puromycin (sc-108071A, Santa Cruz) was added to a final concentration of 5 mg/ml for selection of transduced cells.

Cell conditioned media of cell lines and primary human BCC samples

HeLa cells were allowed to grow to 60-70% confluency, after which, media was replaced with 15mL of fresh media and cells allowed to grow for a further 48 hr. Primary human BCC samples were first dissociated as previously described and single cells were cultured in media for 48 hr. After 48 hr the conditioned media/supernatant were collected and concentrated in Pierce™ concentrators with a molecular weight cut off of 3K MWCO (ThermoFisher Scientific).

ELISA (soluble CD200, CCL4 and soluble FASL)

ELISA's (Supplementary Table 4) were performed following the manufacturer's guidelines, using the cell culture supernatant described earlier. The optical density was measured immediately, using the CLARIOstar plate reader set to 450nm. Wells containing concentrated media of each respective cell types served as the blank.

Human IFN-gamma ELISpot Assay (Interferon-gamma ELISA)

ELISpot assay was used for detecting the secretion of IFN-gamma from NK92MI cells following incubation with $HeLa^{POS}$ and $HeLa^{NEG}$ cell lines. In 6-well tissue culture plates NK92MI cells were incubated with either $HeLa^{POS}$ or $HeLa^{NEG}$ cell lines at a ratio of 1:10, for 4hr. Following incubation, NK92MI cells were removed from the supernatant and added at a concentration of 50,000 cells (100µL/well) per well of a 96-well plate that had been pre-coated with a monoclonal antibody specific for human IFN-gamma. ELISpot assay was then carried out as performed in manufacturers guidelines. The developed microplate was analyzed by

counting the number of spots in each well using the GelCount™ colony counter machine (Oxford Optronix).

Cell viability assay – CellTiter® Glo

Cells were plated (20,000 cells in 100μL) in a white-walled 96-well plate and left overnight to adhere. NK cells were added to the wells at various effector-target ratios. Co-incubations were carried out for 4 hr after which NK cells and media were removed and the adherent tumor cells in wells were washed thoroughly with PBS. Fresh media was added to the wells and an equal amount of CellTiter-Glo® reagent was added to each well (100 μL) and subsequently incubated in the dark at room temperature for 20 mins. Luminescence was recorded using a CLARIOstar plate reader with wells containing only media serving as a blank.

IncuCyte caspase-3/7 NK apoptosis and HeLa killing assays

GFP transduced HeLa cells with and without CD200 expression were seeded into a flat-bottomed white walled 96-well plate at a density of 1,000 cells/well and left to adhere overnight. The next day the caspase-3/7 red reagent was diluted by 1:1000 in complete media to a final concentration of 5μM. Media was aspirated from wells and 100 μL of diluted caspase-3/7 reagent was added to each well along with 5,000 NK92 cells. The plate was then placed into the IncuCyte Live-Cell Analysis System and pictures were taken at 10x magnification every 30 mins for 24 hr. The ability of either $HeLa^{POS}$ or $HeLa^{NEG}$ cells to induce NK cell death could be determined as NK cells undergoing apoptosis turned red with the dye, which could subsequently be measured using the integrated IncuCyte software.

The ability of NK^{POS} cells to induce $HeLa^{POS}$ or $HeLa^{NEG}$ cell death in the presence or absence of exogenous CD200 peptide (4µg/10^6 HeLa cells) was performed by incubating NK and HeLa cells at a ratio of 1:1 in a 96-well white walled plate in the presence or absence of exogenous CD200 peptide. As for the IncuCyte assay, the plate was placed into the IncuCyte Live-Cell Analysis System and pictures were taken at 10x magnification every 30 mins for 24 hr. Since the HeLa cells express GFP, which is maintained after cell division, the IncuCyte software was programmed to measure the levels of HeLa cell death using an image segmentation algorithm to identify individual GFP expressing HeLa cells.

Western Blotting

Samples were diluted in RIPA buffer to equal concentrations. Laemmli buffer (4x) was added to the samples, which were then heated at 95°C for 5 mins. TGX™ FastCast™ premixed acrylamide solutions (BioRad) were used to cast gels. 10ug of protein was loaded into the wells in addition to a molecular weight marker (PageRuler Plus, ThermoFisher), and run at 300V until the desired marker separation was achieved. Trans-Blot® Turbo™ Transfer System (BioRad) was used to transfer protein to the PDVF membrane. Membranes were incubated in blocking buffer of 10% milk or BSA (depending on antibody) in TBST for 1 hour at room temperature. Following incubation, membranes were incubated in the desired primary antibody (Supplementary Table 4) diluted in 5% BSA or milk in TBST and incubated overnight at 4°C on a roller. Following incubation, the membrane was washed in TBST before incubating in horseradish peroxidase (HRP)-conjugated secondary antibody (Abcam) diluted 1:5000 in TBST for 1 hour at room temperature. Membranes are washed again for 4 x 5 mins in TBST before antibody binding was detected by incubating Illumina Forte chemiluminescence reagent (Millipore) on the membrane and imaged using the ChemiDoc MP Imaging System (BioRad).

RNA extraction and cDNA synthesis

RNA was isolated using the Qiagen RNeasy Plus Mini Kit (Qiagen, UK) per manufacturer's instructions. The quality of the extracted RNA was assessed using the Agilent RNA 6000 Nano kit. cDNA synthesis was performed using the Quantitect Reverse Transcription Kit (Qiagen, UK) in 0.2 mL PCR tubes as per manufacturer's guidelines.

Quantitative real-time PCR (qPCR)

For qPCR gene expression studies, all reactions were performed using the TaqMan gene expression assay. Pre-designed TaqMan primer/probes were obtained from Applied Biosystems (Supplementary Table 4). Reactions were run using the TaqMan Universal Master Mix II, with UNG (Applied Biosystems) according to the manufacturer's guidelines. Housekeeping genes (GAPDH and beta actin) were used as reference genes. All reactions were run in three technical replicates. All reactions were run on the QuantStudio 7 Flex Real-Time PCR system (Applied Biosystems) supplemented with the QuantStudio software. Gene expression analysis of qPCR data was analysed using the ΔΔCt method to calculate fold change ($2^{\wedge-\Delta\Delta Ct}$) relative to control.

Bioinformatic analysis

Microarray data were analyzed using the Bioconductor packages in the R statistical program for data processing for all the subsequent steps. For analyzing Illumina microarray data, the 'Lumi' package was used for assessing quality control of the samples. Outliers were removed, before transforming and normalizing the data. Finally, genes were annotated and mapped at the probe level using 'lumiHumanAll.db' and 'luminHumanIDMapping' programs respectively. Genes/probes whose expression was absent between the samples were then filtered out. Next,

we performed comparisons between the treated groups and the untreated group by using the Limma program, again in the Bioconductor package to calculate the level of gene differential expression. A list of differentially expressed genes (DEGs) was obtained at a p value <0.05. Following this we used the expression values of DEGs to perform pathway analysis using Gene Set Enrichment Analysis (GSEA; Broad Institute) (4,5). Fold changes in gene expression were calculated from LogFC values, where fold changes of +1 or –1 indicate no change in gene expression relative to the control.

Analyzing PRECOG (Prediction of Clinical Outcomes from Genomic Profiles) and CIBERSORT (Cell type Identification By Estimating Relative Subsets Of known RNA Transcripts) Datasets

The pan-cancer resource from ~18,000 human tumors and associated overall survival outcomes across 39 malignancies, termed Prediction of Clinical Outcomes from Genomic Profiles (PRECOG; http://precog.stanford.edu) (6). Newman *et al.,* (2015) applied an algorithm, termed Cell-type Identification By Estimating Relative Subsets Of RNA Transcripts (CIBERSORT; http://cibersort.stanford.edu) (7), to the PRECOG resource in order to determine leukocyte representation in bulk tumor transcriptomes, and subsequently identified associations between 22 distinct leukocyte subsets and cancer survival. The significant differentially expressed genes used to identify activated and resting NK cells from 22 immune cell profiles after filtering were: Activated NK cells (*APOBEC3G, APOL6, CCL4, CCL5, CCND2, CD244, CD247, CD69, CD7, CD96, CDK6, CSF2, CST7, CTSW, DPP4, FASLG, GNLY, GPR171, GPR18, GRAP2, GZMA, GZMB, GZMH, GZMM, IFNG, IL12RB2, IL18R1, IL18RAP, IL2RB, KIR2DL1, KIR2DL4, KIR2DS4, KIR3DL2, KLRB1, KLRC3, KLRD1, KLRF1, KLRK1, LCK, LTA, LTB, NAALADL1, NCR3, NKG7, OSM, PRF1, PRR5L, PTGDR, PTGER2, PTPRCAP, PVRIG, S1PR5, SH2D1A, SOCS1, TBX21, TNFSF14,*

TRDC, TXK, ZAP70) and Resting NK cells (*AZU1, BPI, CAMP, CCL5, CD160, CD2, CD244, CD247, CD7, CD96, CDHR1, CEACAM8, CST7, CTSW, DEFA4, ELANE, GFI1, GNLY, GZMA, GZMB, GZMH, GZMK, GZMM, IL12RB2, IL18R1, IL18RAP, IL2RB, KIR2DL1, KIR3DL2, KLRB1, KLRC3, KLRC4, KLRD1, KLRF1, KLRK1, LCK, MGAM, MS4A3, NAALADL1, NKG7, NME8, PLEKHF1, PRF1, PRR5L, PTGDR, PTPRCAP, PVRIG, S1PR5, SH2D1A, TBX21, TEP1, TRBC1, TRDC, TTC38, TXK, ZAP70, ZNF135*). Both PRECOG and immune-PRECOG databases were used to generate data matrices for the associations (in the form of z-scores), between CD200 expression and overall cancer survival outcomes, and leukocyte subsets and overall cancer survival outcomes, respectively. Briefly, PRECOG and immune-PRECOG databases were downloaded and filtered for CD200 expression and NK cell status and rows with NA values were discarded. Normal QQ plot and residual plots were generated to test for normality and variance in the data, respectively. Normality of the data was confirmed, and assumptions of equal variance were met. Finally, simple linear regressions were performed in GraphPad Prism to determine the correlation between immune cell phenotypes and CD200 prognostic z scores in a number of different cancer types. For the z-scores, negative values indicate good prognosis and positive values indicate poor prognosis.

References

1. Morgan H, Olivero C, Patel GK. Identification of human cutaneous basal cell carcinoma cancer stem cells. In: *Methods in Molecular Biology*. Vol 1879. ; 2019:435-450. doi:10.1007/7651_2018_133

2. Olivero C, Morgan H, Patel GK. Identification of human cutaneous squamous cell carcinoma cancer stem cells. In: *Methods in Molecular Biology*. Vol 1879. Humana Press Inc.; 2019:415-433. doi:10.1007/7651_2018_134

3. Lorenzo-Herrero S, Sordo-Bahamonde C, Gonzalez S, López-Soto A. CD107a degranulation assay to evaluate immune cell antitumor activity. In: *Methods in Molecular Biology*. Vol 1884. Humana Press Inc.; 2019:119-130. doi:10.1007/978-1-4939-8885-3_7

4. Subramanian A, Tamayo P, Mootha VK, et al. Gene set enrichment analysis: A knowledge-based approach for interpreting genome-wide expression profiles. *Proc Natl Acad Sci U S A*. 2005;102(43):15545-15550. doi:10.1073/pnas.0506580102

5. Mootha VK, Lindgren CM, Eriksson KF, et al. PGC-1α-responsive genes involved in oxidative phosphorylation are coordinately downregulated in human diabetes. *Nat Genet*. 2003;34(3):267-273. doi:10.1038/ng1180

6. Gentles AJ, Newman AM, Liu CL, et al. The prognostic landscape of genes and infiltrating immune cells across human cancers. *Nat Med*. 2015;21(8):938-945. doi:10.1038/nm.3909

7. Newman AM, Liu CL, Green MR, et al. Robust enumeration of cell subsets from tissue expression profiles. *Nat Methods*. 2015;12(5):453-457. doi:10.1038/nmeth.3337
